# Expansion of Neutrophils and Classical and Nonclassical Monocytes as a Hallmark in Relapsing-Remitting Multiple Sclerosis

**DOI:** 10.3389/fimmu.2020.00594

**Published:** 2020-04-29

**Authors:** David Haschka, Piotr Tymoszuk, Gabriel Bsteh, Verena Petzer, Klaus Berek, Igor Theurl, Thomas Berger, Günter Weiss

**Affiliations:** ^1^Department of Internal Medicine II, Medical University of Innsbruck, Innsbruck, Austria; ^2^Department of Neurology, Medical University of Vienna, Vienna, Austria; ^3^Department of Neurology, Medical University of Innsbruck, Innsbruck, Austria

**Keywords:** neutrophils, classical monocytes, nonclassical monocytes, multiple sclerosis, relapsing-remitting multiple sclerosis

## Abstract

Neutrophils and monocytes encompassing the classical, intermediate, and nonclassical population constitute the majority of circulating myeloid cells in humans and represent the first line of innate immune defense. As such, changes in their relative and absolute amounts serve as sensitive markers of diverse inflammatory conditions. Multiple sclerosis (MS) is a chronic autoimmune disease of the central nervous system, causing demyelination and axonal loss, affecting various neuron functions and often causing irreversible neurological disability. MS disease course is individually highly heterogeneous but can be classified as progressive (PMS) or relapsing-remitting (RRMS). Each MS course type may be further characterized as active or inactive, depending on the recent disability progression and/or current relapses. Data on specific alterations of the myeloid compartment in association with MS disease course are scarce and conflicting. In the current study, we systematically immunophenotyped blood myeloid leukocytes by flow cytometry in 15 healthy and 65 MS subjects. We found a highly significant expansion of granulocytes, CD15^+^ neutrophils, and classical and nonclassical monocytes in inactive RRMS (RRMSi) with concomitant shrinkage of the lymphocyte compartment, which did not correlate with biochemical readouts of systemic inflammation. Each of these leukocyte populations and the combined myeloid signature accurately differentiated RRMSi from other MS forms. Additionally, nonclassical monocyte proportions were particularly elevated in RRMSi individuals receiving disease-modifying therapy (DMT), such as natalizumab. Our results suggest that flow cytometry-based myeloid cell immunophenotyping in MS may help to identify RRMSi earlier and facilitate monitoring of DMT response.

## Introduction

Multiple sclerosis (MS) is a chronic auto-inflammatory disease of the central nervous system (CNS) causing demyelination and axonal loss. Although MS pathophysiology is not completely understood, expansion of myelin-reactive helper T cells is a key feature at the cellular level, orchestrating inflammatory reaction, immune cell infiltration, and neuronal damage (1–3). MS disease course is individually highly heterogeneous and can initially encompass motor, visual, and sensory dysfunction as well as progressing physical disability and cognitive deterioration (1). According to clinical course, MS can be classified as follows: (1) relapsing-remitting MS (RRMS; ~85–90% of new cases) manifesting as periodically occurring neurological symptoms (relapses) followed by clinical remissions and (2) primary progressive MS (PPMS; ~10–15%) characterized by continuous disability progression. Over time, 50–80% of untreated RRMS patients will also develop progressive disability worsening independent of relapses and similar to PPMS termed secondary PMS (SPMS) (1). The current concept of MS pathology includes both inflammation, which is predominant in RRMS, and neurodegeneration, which is considered the key contributor to disability and development of PPMS and SPMS (1, 3). Thus, PPMS and SPMS can be subsumed as PMS (4). Either of these forms can be further classified as momentarily active or inactive, based on relapse frequencies and/or pace of disability progression measured by Expanded Disability Status Scale (EDSS) (1, 5).

The recent update of MS diagnostic guidelines, so-called McDonald criteria, stresses the importance of early diagnosis of MS, possibly at the time of the first MS relapse (5). For RRMS, a rapidly growing number of disease-modifying therapy (DMT) have proven to reduce number of relapses, brain and spinal MS activity determined by magnetic resonance imaging [MRI; new or enlarging T2 lesions or gadolinium-enhanced (GE) T1 lesions] and, to a lesser extent, delay disability progression, whereas efficacy of DMT in PMS is still limited (1, 6). Importantly, RRMS is hallmarked by an extremely variable clinical course both within and between individuals. Whereas, some patients may show highly active and breakthrough disease despite treatment, others may have very mild courses not necessarily requiring DMT (7). Thus, an individualized approach to MS diagnosis, prognosis, and treatment decisions is paramount. However, biomarkers enabling such personalized strategy are lacking, especially those assessing response to DMT at early stages.

In contrast to the well-defined role of T cells in MS pathophysiology, far less is known about the contribution of innate immunity (2). On the one hand, it is postulated that inflammatory myeloid cells, such as blood-borne monocytes or neutrophils, monocyte-derived macrophages, and CNS-resident microglia, participate in blood–brain barrier breakdown (8), perpetuate the inflammation and T cell priming (9–13), and mediate myelin sheath destruction (14–16). On the other hand, microglia (16) or particular subtypes of monocytes (17) and neutrophils (18) were described to exert immunomodulatory functions in MS such as myelin debris removal or suppression of T cell immunity. Owing to their functional relevance, immunophenotyping and quantification of circulating T and B cells as well as of monocytes and granulocytes are regarded as a promising approach in search for potential MS biomarkers (2, 19, 20). In particular, alterations of the monocyte-to-lymphocyte and neutrophil-to-lymphocyte ratio (21), expansion of suppressive and inflammatory neutrophils (18, 22), and changes in relative distribution of monocyte subtypes (23–28) were observed in MS patients and linked to disease activity, degree of disability (EDSS), and administration of DMT.

According to expression of specific surface molecules, cytokine secretion, and phagocytic activity, human monocytes can be classified into the major population of classical monocytes (CD14^++^ CD16^−^) and minor subpopulations of intermediate (CD14^++^ CD16^+^) and nonclassical monocytes (CD14^+^ CD16^+^) (29–31). Alterations of intermediate or nonclassical monocyte levels within the pan-monocyte population are associated with a plethora of inflammatory diseases (30, 32, 33), including MS (24–28). In MS, however, these reports provide partially conflicting data and often investigate only one type of MS disease course or do not differentiate between disease course types.

In the current study, we systematically delved into quantitative changes of circulating myeloid cells in MS patients stratified by disease course type and DMT. We found selective expansion of granulocytes, CD15^+^ neutrophils, and classical and nonclassical monocytes accompanied by reduction of lymphocytes in inactive RRMS (RRMSi) patients as compared with those of healthy controls and other MS forms. Importantly, we demonstrate that quantification of these cell types could be applied for robust discrimination between MS types. In addition, we report elevated levels of nonclassical monocytes in RRMSi patients treated with the monoclonal anti-very late antigen 4 (anti-VLA4; anti-integrin α4β1) antibody natalizumab.

## Results

### Minor Changes in Monocyte Subset Distribution Within HLA-DR^+^ Cells in Multiple Sclerosis Disease Course Types

To quantify granulocytes, neutrophils, and monocyte subsets in blood of healthy controls (*n* = 15) and MS patients (*n* = 65), we established a seven-color flow cytometry staining panel. The first step of our cytometry data analysis enabled identification of circulating lymphoid cells defined by expression of lineage (Lin^+^), that is, T (CD3), B (CD19), and NK (CD56) cell markers, and pan-granulocytes defined as lineage-negative (Lin^−^) and highly granular [high sideward scatter (SSC^hi^)] cells (Supplementary Figure S1). Next, following exclusion of Lin^+^ leukocytes, CD15^+^ neutrophils were defined as CD15 positive, high granularity (SSC^hi^) cells. Pan-monocytes were identified as non-T, B, NK, and non-neutrophil cells expressing HLA-DR and further subdivided into classical, intermediate, and nonclassical cells by CD14 and CD16 expression (33, 34) (Supplementary Figure S1).

**Figure 1:**
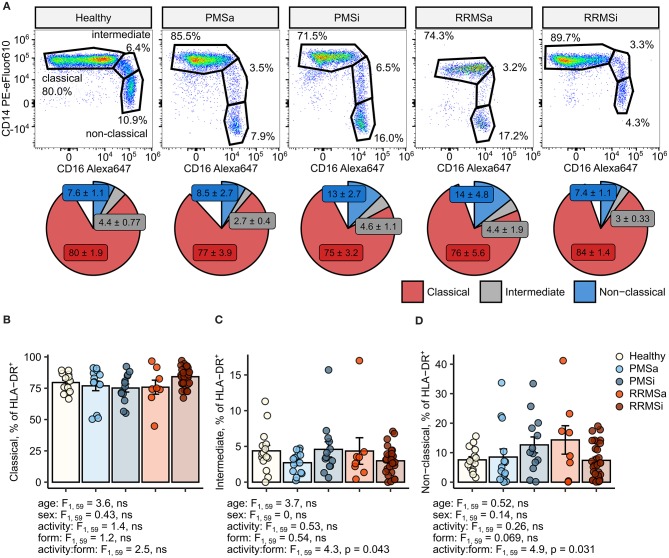
Minor alterations of monocyte subtype distribution pattern in multiple sclerosis (MS) course types. Classical, intermediate, and nonclassical monocytes were identified in whole-blood samples from healthy controls (*n* = 15) and MS patients stratified by disease course type [active progressive MS (PMSa): *n* = 14, inactive progressive MS (PMSi): *n* = 13, active relapsing-remitting MS (RRMSa): *n* = 8, and inactive relapsing-remitting MS (RRMSi): *n* = 30] as presented in Supplementary Figure S1. **(A)** Representative results of CD14 and CD16 staining in CD45^+^ Lineage^−^ CD15^−^ HLA-DR^+^ pan-monocytes are shown. Pie plots display levels of monocyte subtypes expressed as percentage of CD45^+^ Lineage^−^ CD15^−^ HLA-DR^+^ pan-monocytes. Means with SEM are presented. **(B–D)** Levels of monocyte subtypes expressed as percent of CD45^+^ Lineage^−^ CD15^−^ HLA-DR^+^ pan-monocytes. Each point denotes a single observation, bars depict group-wise means, and error bars represent SEM. Statistical significance was determined by one-way (healthy/MS disease status) and two-way (disease progression form, activity and form: activity interaction, MS collective) analysis of covariance (ANCOVA) with age and sex as confounders. Results of the two-way ANCOVA are presented under the plots. *post hoc* testing was performed with Benjamini–Hochberg-corrected two-tailed *T* tests. Significant results of *post hoc* test are presented within the plots. **(B)** Classical monocytes. ANCOVA for the disease status: *F*_1, 76_ = 0.43, ns; age: *F*_1, 76_ = 1.2, ns; sex: *F*_1, 76_ = 0.057, ns. **(C)** Intermediate monocytes. ANCOVA for the disease status: *F*_1, 76_ = 2.7, ns; age: *F*_1, 76_ = 0.45, ns; sex: *F*_1, 76_ = 0.005, ns. **(D)** Nonclassical monocytes. ANCOVA for the disease status: *F*_1, 76_ = 0.13, ns; age: *F*_1, 76_ = 0.8, ns; sex: *F*_1, 76_ = 0.27, ns.

In MS individuals, skewing of the typical pattern of monocyte sub-population distribution within pan-monocytes toward intermediate and nonclassical monocytes was described (24–28). To recapitulate these results, we first assessed the levels of monocyte subsets within the HLA-DR^+^ pan-monocyte population in healthy individuals and MS patients stratified by disease course type and disease activity (Figure 1). This analysis revealed nonsignificantly elevated proportions of classical monocytes in RRMSi patients (Figures 1A,B) and an increase in nonclassical monocytes in inactive PMS (PMSi) and active RRMS (RRMSa) participants [*p* = 0.031 for disease form: activity interaction, analysis of covariance (ANCOVA), Figures 1A,D]. Of note, neither age nor gender, which were included in the ANCOVA models used for data analysis, significantly influenced the proportion of monocyte populations within HLA-DR^+^ cells (Figures 1B–D; see age and sex ANCOVA terms).

### Quantitative Expansion of Pan-Granulocytes, CD15^+^ Neutrophils, and Classical and Nonclassical Monocytes in Inactive Relapsing-Remitting Multiple Sclerosis

**Figure 2:**
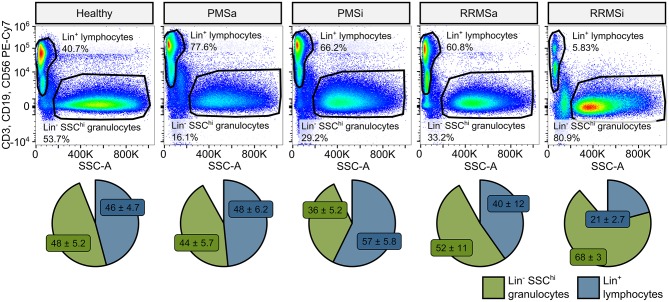
Expansion of granulocytes and shrinkage of the lymphoid compartment in inactive relapsing-remitting multiple sclerosis (RRMSi) individuals. Lineage^+^ lymphocytes and Lineage^−^ SSC^hi^ granulocytes were identified in whole-blood samples from healthy controls (*n* = 15) and MS patients stratified by disease course type [active progressive MS (PMSa): *n* = 14, inactive progressive MS (PMSi): *n* = 13, active relapsing-remitting MS (RRMSa): *n* = 8, and RRMSi: *n* = 30] as presented in Supplementary Figure S1. Representative flow cytometry results with pre-gating for CD45^+^ cells are shown. Pie plots display levels of lymphocytes and granulocytes expressed as percentage of CD45^+^ blood leukocytes. Means with SEM are presented.

**Figure 3:**
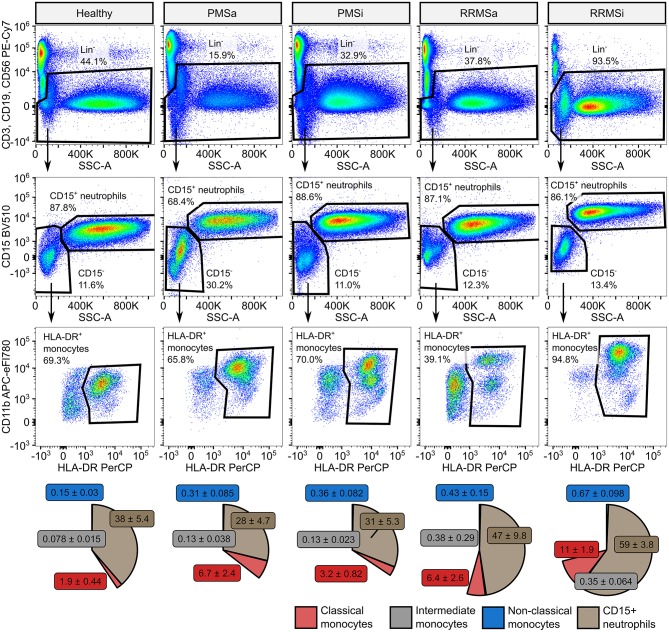
Specific expansion of neutrophil and monocyte compartment in inactive relapsing-remitting multiple sclerosis (RRMSi) individuals. CD15^+^ neutrophils and monocyte subsets were identified in whole-blood samples from healthy controls (*n* = 15) and MS patients stratified by disease course type [active progressive MS (PMSa): *n* = 14, inactive progressive MS (PMSi): *n* = 13, active relapsing-remitting MS (RRMSa): *n* = 8, and RRMSi: *n* = 30] as presented in Supplementary Figure S1. Representative flow cytometry results with pre-gating for CD45^+^ cells are shown. Pie plots display levels of CD15^+^ neutrophils and classical, intermediate, and nonclassical monocytes as percentage of CD45^+^ blood leukocytes. Means with SEM are presented.

**Figure 4:**
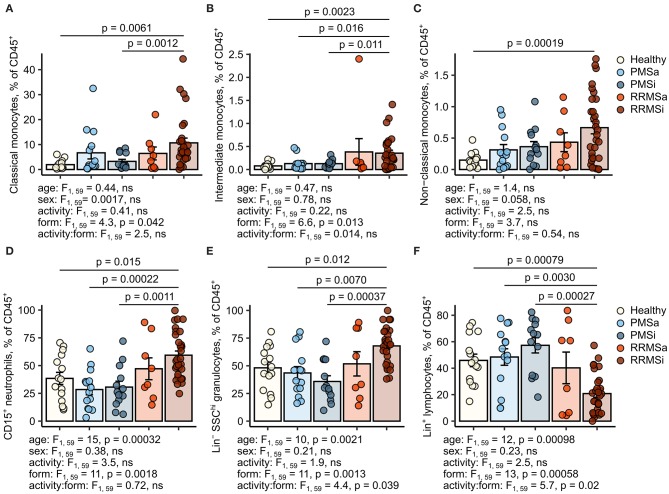
Increased percentages of neutrophils and classical and nonclassical monocytes in inactive relapsing-remitting multiple sclerosis (RRMSi) individuals. Lineage^+^ lymphocytes, Lineage^−^ SSC^hi^ granulocytes, CD15^+^ neutrophils, and monocyte subpopulations were identified in whole-blood samples from healthy controls (*n* = 15) and MS patients stratified by disease course type [active progressive MS (PMSa): *n* = 14, inactive progressive MS (PMSi): *n* = 13, active relapsing-remitting MS (RRMSa): *n* = 8, and RRMSi: *n* = 30] as presented in Supplementary Figure S1. Levels of the studied populations are expressed as percentages of CD45^+^ blood leukocytes. Each point denotes a single observation, bars depict group-wise means, and error bars represent SEM. Statistical significance was determined by one (healthy/MS disease status) and two-way (disease progression form, activity and form: activity interaction, MS collective) analysis of covariance (ANCOVA) with age and sex as confounders. Results of the two-way ANCOVA are presented under the plots. *post hoc* testing was performed with Benjamini–Hochberg-corrected two-tailed *T* tests. Significant results of *post hoc* test are presented within the plots. **(A)** Classical monocytes. ANCOVA for the disease status: *F*_1, 76_ = 5.6, *p* = 0.020; age: *F*_1, 76_ = 0.2, ns; sex: *F*_1, 76_ = 0.54, ns. **(B)** Intermediate monocytes. ANCOVA for the disease status: *F*_1, 76_ = 4.3, *p* = 0.042; age: *F*_1, 76_ = 0.035, ns; sex: *F*_1, 76_ = 0.04, ns. **(C)** Nonclassical monocytes. ANCOVA for the disease status: *F*_1, 76_ = 9.4, *p* = 0.0031; age: *F*_1, 76_ = 0.033, ns; sex: *F*_1, 76_ = 0.36, ns. **(D)** CD15^+^ neutrophils. ANCOVA for the disease status: *F*_1, 76_ = 4.2, *p* = 0.043; age: *F*_1, 76_ = 5.8, *p* = 0.018; sex: *F*_1, 76_ = 1.1, ns. **(E)** Lineage^−^ SSC^hi^ granulocytes. ANCOVA for the disease status: *F*_1, 76_ = 3, ns; age: *F*_1, 76_ = 3.5, ns; sex: *F*_1, 76_ = 0.68, ns. **(F)** Lineage^+^ lymphocytes. ANCOVA for the disease status: *F*_1, 76_ = 4.9, *p* = 0.030; age: *F*_1, 76_ = 3.4, ns; sex: *F*_1, 76_ = 0.74, ns.

**Figure 5:**
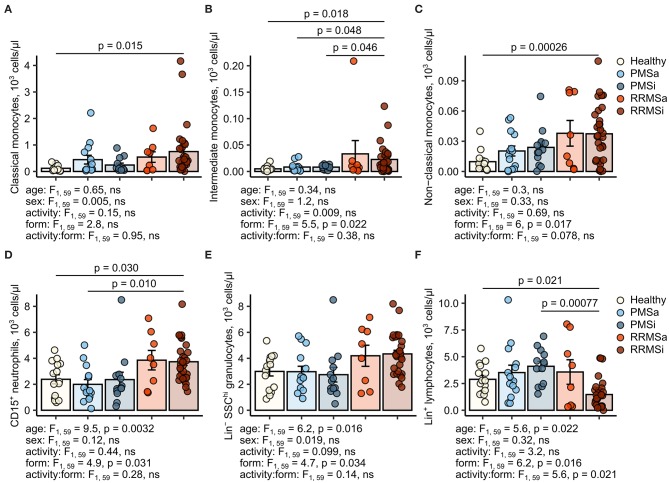
Increased counts of neutrophils and classical and nonclassical monocytes in inactive relapsing-remitting multiple sclerosis (RRMSi) individuals. Lineage^+^ lymphocytes, Lineage^−^ SSC^hi^ granulocytes, CD15^+^ neutrophils, and monocyte subpopulations were identified in whole-blood samples from healthy controls (*n* = 15) and MS patients stratified by disease course type [active progressive MS (PMSa): *n* = 14, inactive progressive MS (PMSi): *n* = 13, active relapsing-remitting MS (RRMSa): *n* = 8 and RRMSi: *n* = 30] as presented in Supplementary Figure S1. Levels of the studied populations are expressed as counts per microliter of whole blood. Each point denotes a single observation, bars depict group-wise means, and error bars represent SEM. Statistical significance was determined by one (healthy/MS disease status) and two-way (disease progression form, activity and form: activity interaction, MS collective) analysis of covariance (ANCOVA) with age and sex as confounders. Results of the two-way ANCOVA are presented under the plots. *post hoc* testing was performed with Benjamini–Hochberg-corrected two-tailed *T* tests. Significant results of *post hoc* test are presented within the plots. **(A)** Classical monocytes. ANCOVA for the disease status: *F*_1, 76_ = 5, *p* = 0.029; age: *F*_1, 76_ = 0.017, ns; sex: *F*_1, 76_ = 0.56, ns. **(B)** Intermediate monocytes. ANCOVA for the disease status: *F*_1, 76_ = 3.6, ns; age: *F*_1, 76_ = 0.007, ns; sex: *F*_1, 76_ = 0.22, ns. **(C)** Nonclassical monocytes. ANCOVA for the disease status: *F*_1, 76_ = 9.8, *p* = 0.0025; age: *F*_1, 76_ = 0.39, ns; sex: *F*_1, 76_ = 0.032, ns. **(D)** CD15^+^ neutrophils. ANCOVA for the disease status: *F*_1, 76_ = 5.8, *p* = 0.019; age: *F*_1, 76_ = 4.8, *p* = 0.032; sex: *F*_1, 76_ = 1.3, ns. **(E)** Lineage^−^ SSC^hi^ granulocytes. ANCOVA for the disease status: *F*_1, 76_ = 4.8, *p* = 0.031, *F*_1, 76_ = 3, ns, *F*_1, 76_ = 0.96, ns. **(F)** Lineage^+^ lymphocytes. ANCOVA for the disease status: *F*_1, 76_ = 0.63, ns; age: *F*_1, 76_ = 1.5, ns; sex: *F*_1, 76_ = 0.23, ns.

During the analysis of flow cytometry data from RRMSi patients, we noticed a drastic reduction of the lymphoid Lin^+^ cell compartment (i.e., CD3^+^ T cells, CD19^+^ B cells, and CD56^+^ NK cells) within circulating pan-leukocytes (Figure 2) paralleled by expansion of Lin^−^ cells including SSC^hi^ granulocytes (Figure 2), CD15^+^ neutrophils, and HLA-DR^+^ pan monocytes (Figure 3). A more detailed analysis of particular myeloid cell populations showed highly elevated CD45^+^ percentages and counts of classical, intermediate, and nonclassical monocytes as well as CD15^+^ neutrophils as compared with those of healthy participants (Figures 4A–D, 5A–D). Among the investigated leukocyte types and classical and intermediate monocytes (Figures 4A,B, 5D,E), SSC^hi^ granulocytes and CD15^+^ neutrophils (Figures 4D,E, 5D,E) were also found to be significantly affected by MS course type, in terms of both relative proportions and absolute counts. For these cells as well as absolute counts of nonclassical monocytes, a specific expansion in RRMS in comparison with PMS was observed with a peak abundance in RRMSi individuals. Additionally, the RRMSi group demonstrated the lowest proportions and counts of lymphoid cells (Figures 4F, 5F).

Importantly, these alterations in myeloid cell pattern and reduction in lymphoid cells in RRMS and, in particular, in RRMSi remained significant even after accounting for differences in age and gender proportions between the study groups (Figures 4, 5, see age and sex terms in ANCOVA, Supplementary Table S1).

### Myeloid Cell Expansion in Inactive Relapsing-Remitting Multiple Sclerosis Is Only Weakly Linked to Systemic Inflammation and Disability Status

RRMS is considered an inflammatory form of MS where elevated levels of either particular neutrophil subtypes (18, 22) or pro-inflammatory cytokines (12, 24, 26) have been reported. We therefore checked if the expansion of the classical and inflammatory monocyte subset and increase in pan-granulocytes and CD15^+^ neutrophils in RRMSi could be linked to hematological and biochemical markers of inflammation.

Canonical inflammatory readouts such as routinely determined complete blood count (CBC) of leukocytes and neutrophils, C-reactive protein (CRP), IL-6, and neopterin serum concentrations were hardly altered between MS course types (Supplementary Figure S2). However, when correlations between the pan-leukocyte percentage of classical monocytes and these inflammatory markers were performed for each individual study group, a borderline significant association between CBC-determined neutrophil counts and the proportion of classical monocytes could be noticed in the RRMSi arm (Supplementary Figure S3). An analogical analysis for nonclassical monocytes revealed, in turn, inverse correlations with the CBC neutrophil and total leukocyte level in PMSi and RRMSi and a similarly negative association between the nonclassical monocyte subset and IL-6 levels in the PMSi group (Supplementary Figure S4). For the pan-leukocyte percentages of neutrophils, there was a trend toward correlation with CBC-determined absolute neutrophil count in PMSi and RRMSa individuals, and, surprisingly, a significant negative association with CBC-determined total leukocyte numbers (Supplementary Figure S5). The later correlation could also be discerned for Lin^−^ SSC^hi^ granulocytes (Supplementary Figure S6). This fairly weak positive correlation pattern of classical monocytes with a single classical inflammatory marker and negative association of nonclassical monocytes with two out of five studied inflammatory readouts lets us conclude that the myeloid cell expansion in RRMSi could not be unequivocally explained by the systemic inflammatory status of the patient.

Myeloid cell expansion represented by elevated neutrophil-to-lymphocyte and monocyte-to-lymphocyte ratios in MS patients was reported to correlate with disability status (21). Hence, we studied possible associations between the CD45 percentages and numbers of Lin^−^ SSChi granulocytes, CD15^+^ neutrophils, and classical and nonclassical monocytes and EDSS in our MS cohort. As presented in Supplementary Figure S7, a significant correlation between the pan-leukocyte proportions of classical monocytes and the disability score was detected in RRMSi. For all other investigated leukocyte populations and study groups, no association with EDSS was discerned.

### Levels of Neutrophils and Classical and Nonclassical Monocytes Serve as Markers of Inactive Relapsing-Remitting Multiple Sclerosis

**Figure 6:**
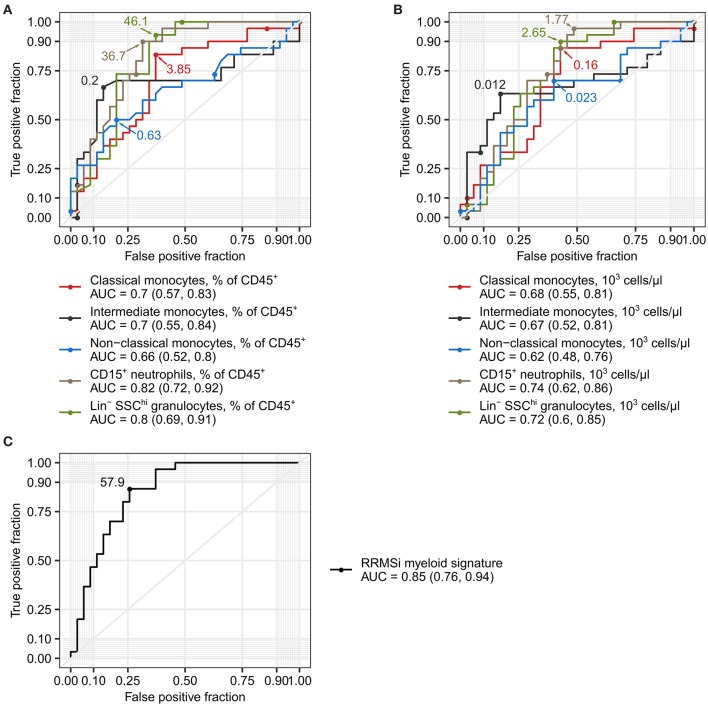
Levels of neutrophils and classical and nonclassical monocytes as inactive relapsing-remitting multiple sclerosis (RRMSi) markers. Lin^+^ lymphocytes, Lin^−^ SSC^hi^ granulocytes, CD15^+^ neutrophils, and monocyte subpopulations were identified and quantified in MS study participants stratified by disease course type [active progressive MS (PMSa): *n* = 14, inactive progressive MS (PMSi): *n* = 13, active relapsing-remitting MS (RRMSa): *n* = 8, and inactive relapsing-remitting MS (RRMSi): *n* = 30] as presented in Supplementary Figure S1. Myeloid signature was calculated as an optimally weighted sum of percentages of neutrophils and classical and nonclassical monocytes within CD45^+^ pan leukocytes. Receiver operating curves (ROCs) display sensitivity and specificity of each parameter as a marker to differentiate between RRMSi and other MS disease courses. In the plots, optimal parameter cutoffs are displayed. In the legends, values of areas under the curve (AUCs) for each ROC are presented with the 95% CI in parentheses. **(A)** Levels of myeloid cell populations and Lin^+^ lymphocytes expressed as percentage of CD45^+^ cells. **(B)** Levels of myeloid cell populations and Lin^+^ lymphocytes expressed as count per microliter of whole blood. **(C)** Myeloid signature.

Disease course prediction and DMT choice in early MS with varying relapse rates and no EDSS progression can be challenging and may be associated with a broad margin of uncertainty (1, 5, 6). Given the profound alterations in the myeloid cell compartment in RRMSi patients (Figures 3–5), we asked whether the levels of circulating Lin^−^ SSC^hi^ granulocytes, CD15^+^ neutrophils, and monocyte subsets can be used as diagnostic markers to discriminate between MS course types. As demonstrated in Figure 6A, abundance of these leukocyte populations in the peripheral blood expressed as percentage of CD45^+^ pan-leukocytes demonstrated substantial discriminatory power for RRMSi detection in the mixed MS cohort. The correctness of discrimination calculated as area under the curve (AUC) of receiver operating curve (ROCs) ranged from 66% for nonclassical monocytes to 82% for CD15^+^ neutrophils (Figure 6A, Supplementary Table S2). For the predictive potential of CD15^+^ neutrophils, the optimal cutoff value was a relative percentage of 36.7%, which displayed 90% sensitivity and 69% specificity (Figure 6A, Supplementary Table S2). Similarly, a discriminatory potential for the diagnosis of RRMSi was found for the absolute counts of classical and intermediate monocytes, CD15^+^ neutrophils, and Lin^−^ SSC^hi^ granulocytes (Figure 6B, Supplementary Table S2).

Next, we asked if a combination of the best-performing RRMSi markers, that is, percentages of CD15^+^ neutrophils and classical and nonclassical monocytes within CD45^+^ leukocytes, may further augment the accuracy of the MS course type diagnosis. To obtain such optimal parameter combination, we modeled the RRMSi risk as a function of these parameters with logistic regression. The fitted exponentiated model estimates for CD15^+^ neutrophil and classical and nonclassical monocyte percentages were utilized to calculate a compound parameter, which was termed myeloid signature (see Materials and Methods for details). The myeloid signature demonstrated indeed superiority over the use of single parameters for RRMSi diagnosis with an accuracy of 85% (CI: 76–94%), 87% sensitivity, and 74% specificity at its optimal cutoff value of 57.9 (Figure 6C, Supplementary Table S2). Cumulatively, these data suggest that flow cytometry-based quantification of neutrophils and monocyte subsets, as either single parameters or in a combination, can help in predicting the course of MS.

### Disease-Modifying Therapy in Inactive Relapsing-Remitting Multiple Sclerosis Correlates With Elevated Nonclassical Monocyte Levels

**Figure 7:**
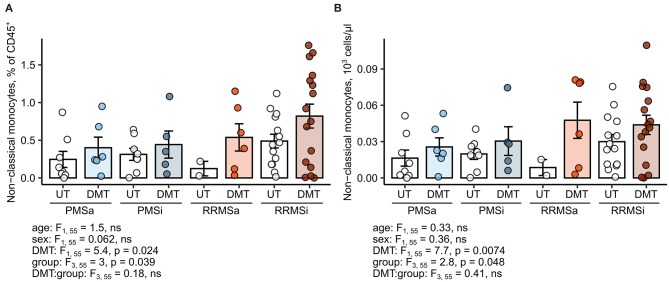
Expansion of nonclassical monocytes in disease-modifying therapy-treated multiple sclerosis (MS) patients. Nonclassical monocytes were identified in blood samples of MS patients, from whom data on disease-modifying therapy [untreated (UT) and disease-modifying therapy (DMT)] were available [UT active progressive MS (PMSa): *n* = 8, DMT PMSa: *n* = 6, UT inactive progressive MS (PMSi): *n* = 8, DMT PMSi: *n* = 5, UT active relapsing-remitting MS (RRMSa): *n* = 5, DMT RRMSa: *n* = 6, UT inactive relapsing-remitting MS (RRMSi): *n* = 14, and DMT RRMSi: *n* = 16] as presented in Supplementary Figure S1. Each point denotes a single observation, bars depict group-wise means, and error bars represent SEM. Statistical significance was calculated with two-way analysis of covariance (ANCOVA) (terms: study group, DMT and group: DMT interaction) with age and sex as confounders. Results of the two-way ANCOVA are presented under the plots. *post hoc* testing was performed with Benjamini–Hochberg-corrected two-tailed *T* tests. Significant results of *post hoc* test are presented within the plots. **(A)** Levels of nonclassical monocytes expressed as percentage of CD45^+^ blood leukocytes. **(B)** Levels of nonclassical monocytes expressed as counts per microliter of whole blood.

Most of the current DMT interfere with leukocyte trafficking pathways, modulate, or deplete a particular cell type at the systemic level (2, 6). Therefore, we sought to investigate if DMT in general and specific drugs in particular impact on the abundance of neutrophils and monocyte subsets. Importantly, samples of MS study participants were obtained prior to steroid drug therapy. First, we found no effect of DMT administration on the monocyte subset distribution pattern; that is, relative percentages of classical, intermediate, and nonclassical cells within HLA-DR^+^ monocytes were not altered (Supplementary Figure S8). Second, no DMT-specific alterations in pan-leukocyte percentages (Supplementary Figure S9) and absolute counts (Supplementary Figure S10) could be detected for classical monocytes, intermediate monocytes, CD15^+^ neutrophils, or Lin^−^ SSC^hi^ granulocytes. In turn, the levels of the nonclassical monocyte subset, both expressed as percent of pan-leukocytes and absolute number, were significantly upregulated in DMT-treated MS patients (ANCOVA for DMT: *p* = 0.024 for percentages and *p* = 0.0074 for counts) and peaked in DMT-treated RRMSi individuals (Figure 7).

**Figure 8:**
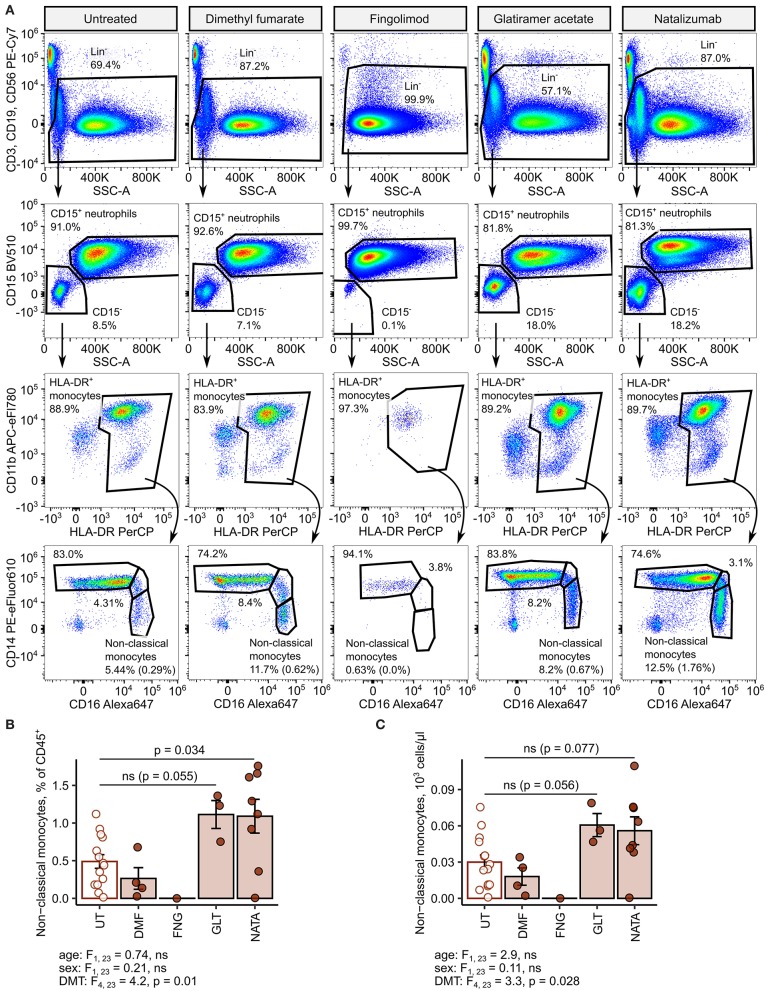
Increased amounts of nonclassical monocytes in natalizumab-treated inactive relapsing-remitting multiple sclerosis (RRMS) patients. Nonclassical monocytes were identified in blood samples of inactive RRMS patients stratified by the kind of disease-modifying therapy [untreated (UT): *n* = 14, dimethyl fumarate (DMF): *n* = 4, fingolimod (FNG): *n* = 1, glatiramer acetate (GLT): *n* = 3, and natalizumab (NATA): *n* = 8] as presented in Supplementary Figure S1. Each point denotes a single observation, bars depict group-wise means, and error bars represent SEM. Statistical significance was calculated with one-way analysis of covariance (ANCOVA) with age and sex as confounders. ANCOVA results are presented under the plots. *post hoc* testing was performed with Benjamini–Hochberg-corrected two-tailed *T* tests. Significant results of *post hoc* test are presented within the plots. **(A)** Representative flow cytometry results with pre-gating for CD45^+^ cells. For nonclassical monocytes, numbers in parentheses indicate the percentage within CD45^+^ pan-leukocytes. **(B)** Levels of nonclassical monocytes expressed as percentage of CD45^+^ blood leukocytes. **(C)** Levels of nonclassical monocytes expressed as counts per microliter of whole blood.

Next, we delved into nonclassical monocyte levels in the RRMSi study group stratified by the DMT drug type. Administration of the anti-VLA4 monoclonal antibody natalizumab resulted in a significant increase of CD45 proportions and nearly significant rise in absolute counts of the nonclassical monocyte subset (Figure 8). Similarly, substantially elevated percentages and counts of these cells could be discerned in the group treated with the myelin-peptide mimic glatiramer acetate (Figure 8).

### Expansion of the Myeloid Cell Compartment Is Observed in Disease-Modifying Therapy-Untreated Inactive Relapsing-Remitting Multiple Sclerosis Patients

As presented above, DMT may influence myeloid cell composition in MS. We questioned, hence, if analogous trends toward expansion of pan-granulocytes, CD15^+^ neutrophils, and classical and nonclassical monocytes could be detected in the momentarily DMT-untreated MS collective.

As shown in Supplementary Figure S11, CD45 percentages of classical monocytes, CD15^+^ neutrophils, and Lin^−^ SSC^hi^ granulocytes were significantly affected by disease course type (PMS/RRMS, ANCOVA: *p* = 0.050, *p* = 0.017, and *p* = 0.0038, respectively) in the DMT-untreated subpopulation with maximal amounts detected in RRMSi patients. The same regulation by the MS course type could be observed for absolute counts of pan-granulocytes and CD15^+^ neutrophils (Supplementary Figure S12). Expansion of these cell types was, like in the whole MS collective, paralleled by a drop in relative levels and numbers of Lin^+^ lymphoid cells (Supplementary Figures S11F, S12F).

Finally, we investigated if the altered myeloid cell distribution pattern may again be utilized for distinction of RRMSi individuals in a mixed disease course collective consisting of momentarily DMT-untreated patients. The results of ROC analysis reveal that pan-leukocyte percentages and numbers of CD15^+^ neutrophils and Lin^−^ SSC^hi^ granulocytes could indeed discriminate between RRMSi and other disease forms also in this setting with an overall diagnose accuracy ranging from 74 to 87% (AUC values, Supplementary Figures S13A,B, Supplementary Table S2). Interestingly, the values of myeloid signature re-calculated for the DMT-untreated participant subpopulation were found to distinguish RRMSi from other MS courses with a larger predictive power than in the entire MS collective [AUC with 95% CI: 0.92 (0.82, 1), sensitivity of 86%, and specificity of 83% at the optimal cutoff, Supplementary Figure S13C, Supplementary Table S2. In summary, we demonstrate that expansion of the myeloid leukocyte compartment takes place in RRMS and, in particular, in RRMSi, and also in DMT-untreated patients and that myeloid cell immunophenotyping may aid at disease course prediction also in such MS individuals.

## Discussion

In the current cross-sectional study, we quantified major circulating myeloid cell populations in MS patients stratified by disease course type and recent disease activity. As such, our study represents an important contribution to the currently ongoing discussion on alterations of the myeloid compartment in MS. A set of studies focusing on changes in monocyte subset levels report either an expansion of classical (23) and CD14^++^ CD16^+^ bona-fide intermediate monocytes (26) or of both intermediate and nonclassical cells (28) or a sole increase in nonclassical monocytes (27) in MS patients. In contrast, a paper by Waschbisch and et al. provided evidence for reduction of the pan-monocyte percentage of the intermediate and nonclassical subset in untreated RRMS subjects (24). In light of these conflicting findings, a few shortages of the previous studies have to be stressed, such as analysis of a mixed MS cohort without distinction between progression forms (26) or disease activity (24) and analysis of pre-purified, monocyte-enriched blood fractions, like peripheral blood mononuclear cells (PBMCs) (23, 27, 28) or CD14^+^ leukocyte fraction (26). We addressed these limitations by (1) analyzing minimally manipulated biological material (red blood cell-lysed EDTA blood), hence reducing bias introduced by some cell enrichment methods; (2) concomitant quantification of multiple myeloid cell types with a comprehensive flow cytometry panel; and (3) investigation of a well-characterized patient population encompassing both RRMS and PMS individuals stratified by the recent disease activity. In our setting, identification of monocytes was accomplished by HLA-DR positivity with exclusion of lymphocytes and neutrophils, which express monocyte subset markers like CD14 and CD16 (33, 34). Applying this strategy, we were not able to identify significant alterations in the subset composition of the HLA-DR^+^ pan-monocyte population, although trends toward increased classical monocytes in RRMSi and nonclassical monocyte in PMSi and RRMSa became evident.

Instead, we could detect a dramatic increase in levels of myeloid cells in RRMS participants culminating in the RRMSi study arm. In particular, both pan-leukocyte percentages and counts of Lin^−^ SSC^hi^ granulocytes, CD15^+^ neutrophils, and classical and nonclassical monocytes were significantly elevated in this group as compared with PMS patients and healthy individuals. Of note, at the same time, the relative abundance and counts of Lin^+^ lymphoid cells in RRMSi were reduced to levels lying far below healthy controls and PMS patients. Hence, we postulate that the expansion of myelocytes in RRMSi may involve not only increased production and/or increased life span of myelocytes but also active depletion of lymphoid cells from circulation, for example, by retention in lymphoid organs. Notably, an analogous skewing of leukocyte distribution could be discerned in the DMT-untreated MS patient subgroup, which indicates that these partly drastic alterations could not be attributed to any specific therapy. Importantly, our results are in line with observations made in the animal model of MS, experimental autoimmune encephalomyelitis (EAE) (8–11, 16, 35) and another form of inflammatory neurodegeneration in humans, amyotrophic lateral sclerosis (ALS) (36). Similarly, elevated levels of neutrophils in blood of MS subjects were described before in few reports (18, 22).

Literature evidence indicates that gender, age, and overall physical status (frailty) may impact on proportions and numbers of circulating myeloid cells including the cell types differentially expanded in RRMSi in our study, like granulocytes (37, 38) and, intermediate and nonclassical monocytes (37–41). In the investigated cohort, differences in age and grade of disability (EDSS) between the MS groups were evident (Supplementary Table S1) and could be in large part explained by the natural course of the disease (1). Bearing this in mind, we included the effects of age and sex in ANCOVA, and the data analysis method of choice. Importantly, in such a setting, the disease course type still proved to significantly affect CD45^+^ percentages and numbers of monocyte subsets, CD15^+^ neutrophils, pan-granulocytes, and lymphocytes, even though age was found to be a significant confounder for some leukocyte types (CD15^+^ neutrophils, granulocytes, and lymphocytes, Figures 4D–F, 5D–F). Concerning the effect of disability on myeloid cells, we could observe a sole, fairly weak positive correlation of EDSS with the CD45^+^ proportions of classical monocytes in the RRMSi group only. Additionally, assuming an increase in myeloid cell numbers with age and worsening in the physical status described in the literature, elevated amounts of circulating monocytes and granulocytic cells are expected in the significantly older PMS groups displaying higher EDSS values. However, the opposite was found in our study. Cumulatively, age and EDSS are unlikely to contribute to the observed expansion of monocytic and granulocytic cells in RRMS and, in particular, in its inactive form.

The question of whether the observed accumulation of granulocytes, CD15^+^ neutrophils, and classical and nonclassical monocytes in RRMSi reflects an ongoing inflammatory process or is rather a sign of immunomodulation in relapsing RRMS individuals could not be unequivocally answered with our data. Naegele et al. (22) found that neutrophils from RRMS patients exhibit a prolonged life span and an inflammatory immune phenotype. Data from animal models and human MS studies suggest that circulating monocytes and neutrophils act as a source of inflammatory cytokines (10, 12, 24) and participate in axonal destruction (8, 9, 11, 15, 16, 35, 42). However, the same cells or specific subsets of classical monocytes and neutrophils, such as HLA-G-positive cells (17) or granulocyte myeloid-derived suppressor cells (G-MDSCs) (18), were shown to counteract priming and expansion of pathogenic T cells. In our study, we could not detect any consistent pro-inflammatory or anti-inflammatory link between canonical markers of systemic inflammation like leukocyte numbers or IL-6 concentrations and the expanding myeloid leukocytes. Similarly, only for classical monocytes but not for other populations increased in RRMSi was a weak correlation with the degree of disability detected. In sum, we postulate the expansion of neutrophils and classical and nonclassical monocytes to be an RRMSi intrinsic phenomenon not coupled with overt systemic inflammation or clinical signs of neurodegeneration.

In addition, we could demonstrate that one of the leukocyte populations accumulating in RRMSi, nonclassical monocytes, was further upregulated in DMT-receiving patients. More specifically, this effect was most prominent in individuals treated with the anti-VLA4 antibody natalizumab and the myelin-peptide mimic glatiramer acetate. Similar effects of those drugs on the nonclassical monocyte population have been reported (24, 26, 28). The main mode of action of natalizumab is blockage of lymphocyte entry into the CNS (2, 6). However, its impact on trafficking of other cells, like egress of neutrophil precursors and erythroblasts, is known (43). Interestingly, Waschbisch et al. demonstrated abilities of human classical monocytes to cross an epithelial barrier in a blood–brain barrier model *in vitro* (24). In this context, it is tempting to speculate whether the increased pan-leukocyte frequency of nonclassical monocytes upon natalizumab exposure results from inhibition of the CNS entry of those cells or their precursors, classical and intermediate monocytes (44).

The latest revision of McDonald criteria stress the necessity of early diagnosis, classification, and therapy of new MS cases, hence justifying the search for novel disease markers (5, 6, 45, 46). Apart from MRI-based and epidemiological disease hallmarks, attempts are made to link immune activation markers in blood and CSF with particular disease forms (21–23, 27, 47, 48), relapse or disability progression (20, 21), and therapy response (2, 23, 25, 28). Here, we demonstrate the usefulness of a simple flow cytometry-based quantification of circulating granulocytes, CD15^+^ neutrophils, and monocyte subsets in prediction of RRMSi. The best-performing parameters, pan-leukocyte percent of CD15^+^ neutrophils, and the myeloid signature (being a linear combination of CD15^+^ neutrophil, classical, and nonclassical monocyte levels), demonstrated an accuracy exceeding 80% in discrimination between RRMSi and other MS forms. Interestingly, the same marker enabled an even more robust RRMSi distinction in momentarily DMT-untreated MS individuals. This suggests that such a minimally invasive technique may be applied, together with the canonical MS monitoring toolbox, to improve diagnosis and to timely initiate DMT. Importantly, myeloid cell immunophenotyping may support early identification of RRMSi and thus assist in optimizing DMT monitoring (1, 5). Whether the expansion of neutrophils and the classical and nonclassical monocyte subset could be linked to early disease progression or relapse frequency warrants further investigation in a prospective setting. Additionally, it would be interesting to study if changes in the myeloid cell distribution pattern correlate with treatment response determined by other “hard” parameters such as counts of CNS lesions and frequency of adverse events. Such study with a larger patient cohort could also account for other immune profile-modifying variables such as body mass index (BMI) or motor performance (49).

MS disease course is both highly variable and unpredictable among individuals, especially in terms of future risk of disability progression. The broad armamentarium of available DMT comprises, on the one hand, highly efficient drugs such as natalizumab, fingolimod, and alemtuzumab, which bear substantial risk of progressive multifocal leukoencephalopathy (PML) or secondary autoimmunity and, on the other, moderately efficient options with favorable long-term safety profiles such as interferon beta, glatiramer acetate, dimethyl fumarate, and teriflunomide. Although the progress achieved in the overall prognosis of MS through the development of DMT is substantial and undebatable, “hit hard and early” is not necessarily the best strategy in every patient, and initial “watchful waiting” after establishment of the diagnosis and ultimately not applying DMT might be a viable option in a proportion of RRMS patients, demonstrating certain features of favorable prognosis (7, 50). Under these circumstances, there is a strong need for reliable biomarkers differentiating active and inactive MS. Blood leukocyte immunophenotyping as proposed in our study might in future append to the expanding tool set of markers of MS progression and therapy response. At the same time, we have to stress that such an approach is certainly a long way from an established biomarker in this regard; and, hence, no treatment decision, especially about continuation of DMT, can be made based on it. However, further research in this promising direction, especially in a prospective setting, is definitely warranted.

Finally, we would like to point out some shortages of the study design. First of all, the cross-sectional nature of the study and a mixed-therapy MS collective may be regarded as a major drawback. However, the current study cohort probably reflects well an average real-life MS population found in most clinical centers. We have to underline that inclusion of completely DMT-naïve MS individuals would be challenging owing to the size of the local MS population and fixed time interval of the study. Another potential limitation is the lacking distinction between PPMS and SPMS patients. Taking into account similar pathomechanisms acting in both PMS forms such as predominant neurodegeneration and localized, microcompartmentalized inflammation (4) and a very low number of available primary PMS cases, we decided to analyze those two groups together.

In summary, our results of systematic quantification of circulating myeloid cells in MS patients stratified by the disease course type and disease activity demonstrate significant accumulation of neutrophils and classical and nonclassical monocytes in RRMSi. Of practical importance, these alterations of the myeloid compartment may be diagnostically utilized for robust discrimination of MS course types and possibly assist in optimizing DMT monitoring.

## Materials and Methods

### Study Approval and Ethics Statement

The study was approved by the local ethics board at the Medical University of Innsbruck (approval number: AM3743-281/4.3). The study was performed in accordance with the Declaration of Helsinki and federal and European regularities and data policy. Each study participant gave written informed consent before recruitment. Participant data were analyzed and stored in anonymous form. Raw data, study results, and the manuscript were made available to all study authors.

### Study Design

Study participants (15 healthy controls and 70 MS patients) were consecutively recruited between April 2017 and May 2018 from the MS clinic of the Department of Neurology at the Medical University of Innsbruck. MS patients aged 18–70 years were classified as follows: (1) active PMS (PMSa; PPMS or SPMS, EDSS progression within the last 6 months before study onset), (2) PMSi (PPMS or SPMS, no EDSS progression within the last 6 months before study onset), (3) RRMSa (a relapse within the last 3 months before the study onset), or (4) RRMSi (no relapse within the last 3 months before the study onset) according to the 2017 McDonald criteria and Lublin criteria (4, 5). EDSS progression was defined as an increase by ≥1.0 point in patients with a baseline score of ≤5.5 or an increase by ≥0.5 points in patients with a baseline score of >5.5. Exclusion criteria were as follows: acute infection/inflammation defined as body temperature > 38.0°C within the last 14 days before sampling and CRP > 1 mg/dl, ongoing interferon therapy, malignancies, and diabetes defined by HbA1c exceeding 47 nmol/mol. Study inclusion was done prior to corticosteroid therapy; none of the MS patients received steroid drug treatment at sampling. Flow cytometry data were available for a total of 15 healthy controls and 65 MS participants. Detailed study cohort characteristics are presented in Supplementary Table S1.

### Flow Cytometry and Inflammatory Marker Determination

For flow cytometry measurement, whole samples of EDTA blood were collected, depleted from red blood cells by lysis with ACK buffer (150 mM of NH_4_Cl, 10 mM of KHCO_3_, and 0.1 mM of Na_2_EDTA), and antibody stained essentially as described (31, 51). The following antibodies were used: PE-Cy7 anti-CD3 (clone OKT3), PE-eFluor610 anti-CD14 (M5E2), Alexa 647 anti-CD16 (3G8), Brilliant Violet 510 (BV510), anti-CD15 (HI98), PE-Cy7 anti-CD19 (HIB19), PE-Cy7 anti-CD56 (CMSSB), and PerCP anti-HLA-DR (TU36). The antibody staining mix was supplemented with 2% rat serum to block unspecific binding by IgG receptors. Samples were measured with a Gallios flow cytometer (Beckman Coulter). Primary flow cytometry data analysis (gating and cell type quantification) was performed with FlowJo version 10 (Becton-Dickinson). Absolute cell counts for a particular flow cytometry-identified population were calculated as a product on its percent within CD45^+^ cells in the sample and total leukocyte numbers determined by CBC as described below.

Serum IL-6 was determined with an electro-chemiluminescence immunoassay (ECLIA), CRP with an immunoturbidimetric assay, and neopterin with an ELISA; and total leukocyte and neutrophil numbers were determined by standard automatic CBC measurement at the Central Institute for Medical and Chemical Diagnostics (ZIMCL), Tirol Kliniken GmbH.

### Predictive Power of Inactive Relapsing-Remitting Multiple Sclerosis Markers, Receiver Operating Curve Modeling

ROC modeling and generalized linear modeling (GLM) were performed with the R programming suite (version 3.6). For calculation of the myeloid signature Score (Figure 6C, Supplementary Figure S13C), the categorical RRMSi disease indicator (1 for RRMSi patients, 0 for other MS patients) was modeled as a function of percent classical monocytes, nonclassical monocytes, and neutrophils within CD45^+^ cells with logistic regression (GLM, logit link function). Myeloid signature was calculated with the following formula:

$$\begin{array}{l} \text{Myeloid Signature }=\text{ exp}\alpha +\text{exp}\beta_{\text{class}}\times \text{Classical}+\text{exp}\beta_{\text{nonclass}}\\ \;\times \text{Nonclassical}+\text{exp}\beta_{\text{neutro}}\times \text{Neutrophils} \end{array}$$

where α stands for the logistic curve intercept; β stands for logit coefficients of the logistic curve; Classical, Nonclassical, and Neutrophils stand for percentages of classical monocytes, nonclassical monocytes, and CD15^+^ neutrophils within CD45^+^ blood cells. The exponentiated estimate values for the whole MS collective were as follows: expα = 0.14, expβ_class_ = 1.1, expβ_nonclass_ = 4.4, and expβ_neutro_ = 1.1. The exponentiated estimate values for the DMT-untreated MS collective were as follows: expα = 0.0016, expβ_class_ = 1.1, expβ_nonclass_ = 25.6, and expβ_neutro_ = 1.1. In both collectives, the complete models predicted the RRMSi risk better than the null model as assessed with likelihood ratio test (LRT; Supplementary Table S3). Inclusion of the classical monocyte term and nonclassical monocyte term significantly improved the model performance in the whole MS collective (*p* = 0.0051, Supplementary Table S3) and nearly significantly improved the model performance in the DMT-untreated MS collective (*p* = 0.072, Supplementary Table S3) in comparison with a nested model containing the neutrophil term only.

ROCs were plotted with the PlotROC package. AUCs and optimally differentiating marker values were calculated using the Youden method using the OptimalCutpoints package.

### Statistics

Statistical analysis and data visualization were performed with the R programming suite (version 3.6) and the tidyverse package bundle (data transformation and plotting). Unless otherwise indicated, data are visualized as bar plots with error bars coding for group-wise mean with SEM and single observations depicted as points. *p* values < 0.05 were considered significant. Additionally, near-significant *p* values are displayed in plots as well (0.05 ≤ *p* < 0.10).

Statistical significance for differences between healthy controls and MS patients were assessed with one-way ANCOVA with age and sex as confounders. Statistical significance for differences attributed to disease course form (PMS/RRMS) and disease activity (active/inactive) and interaction thereof was assessed with two-way ANCOVA with age and sex as confounders. *Post hoc* testing was accomplished with Benjamini–Hochberg-corrected two-tailed *T* tests.

Statistical significance for correlations between two variables were determined with mixed-effect linear models with sex and factorized age (18–30, 30–50, and 50–70 years) as random components. Degrees of freedom for model components were calculated with the Welch–Satterthwaite method. *p* values for linear model estimates (β, β ≠ 0) were calculated with two-tailed *T* test and corrected for multiple comparisons with the Benjamini–Hochberg method. Mixed-effect modeling tasks were performed with the lme4 and lmerTest packages.

Validity of test/model assumptions (normality of variables, normality of residuals, and equal variance of residuals) was visually assessed with quantile–quantile plots and, additionally, with Shapiro–Wilk and Levene tests.

## Data Availability Statement

All datasets generated for this study are included in the article/Supplementary Material as Supplementary Table S4.

## Ethics Statement

The studies involving human participants were reviewed and approved by Ethics Board at the Medical University of Innsbruck. The patients/participants provided their written informed consent to participate in this study.

## Author Contributions

DH and GB designed the study, recruited participants, analyzed biological material, performed data analysis, and wrote the manuscript. PT designed the study, analyzed biological material, performed data analysis, and wrote the manuscript. VP analyzed biological material. KB designed the study and recruited participants. IT and TB wrote the manuscript. GW designed the study and wrote the manuscript.

## Conflict of Interest

GB has participated in meetings sponsored by and received speaker honoraria or travel funding from Biogen, Merck, Novartis, Roche, Sanofi-Genzyme, and Teva and received honoraria for consulting from Biogen, Roche, and Teva. TB has participated in meetings sponsored by and received honoraria (lectures, advisory boards, and consultations) from pharmaceutical companies marketing treatments for MS: Allergan, Bayer, Biogen, Bionorica, Celgene, MedDay, Merck, Novartis, Octapharma, Roche, Sanofi-Genzyme, and Teva. The remaining authors declare that the research was conducted in the absence of any commercial or financial relationships that could be construed as a potential conflict of interest.
