# Supplementary figures and images for: Expansion of Neutrophils and Classical and Nonclassical Monocytes as a Hallmark in Relapsing-Remitting Multiple Sclerosis
Source: Front Immunol. 2020 Apr 29;11:594. doi: 10.3389/fimmu.2020.00594 (PMC7202453; doi:10.3389/fimmu.2020.00594)

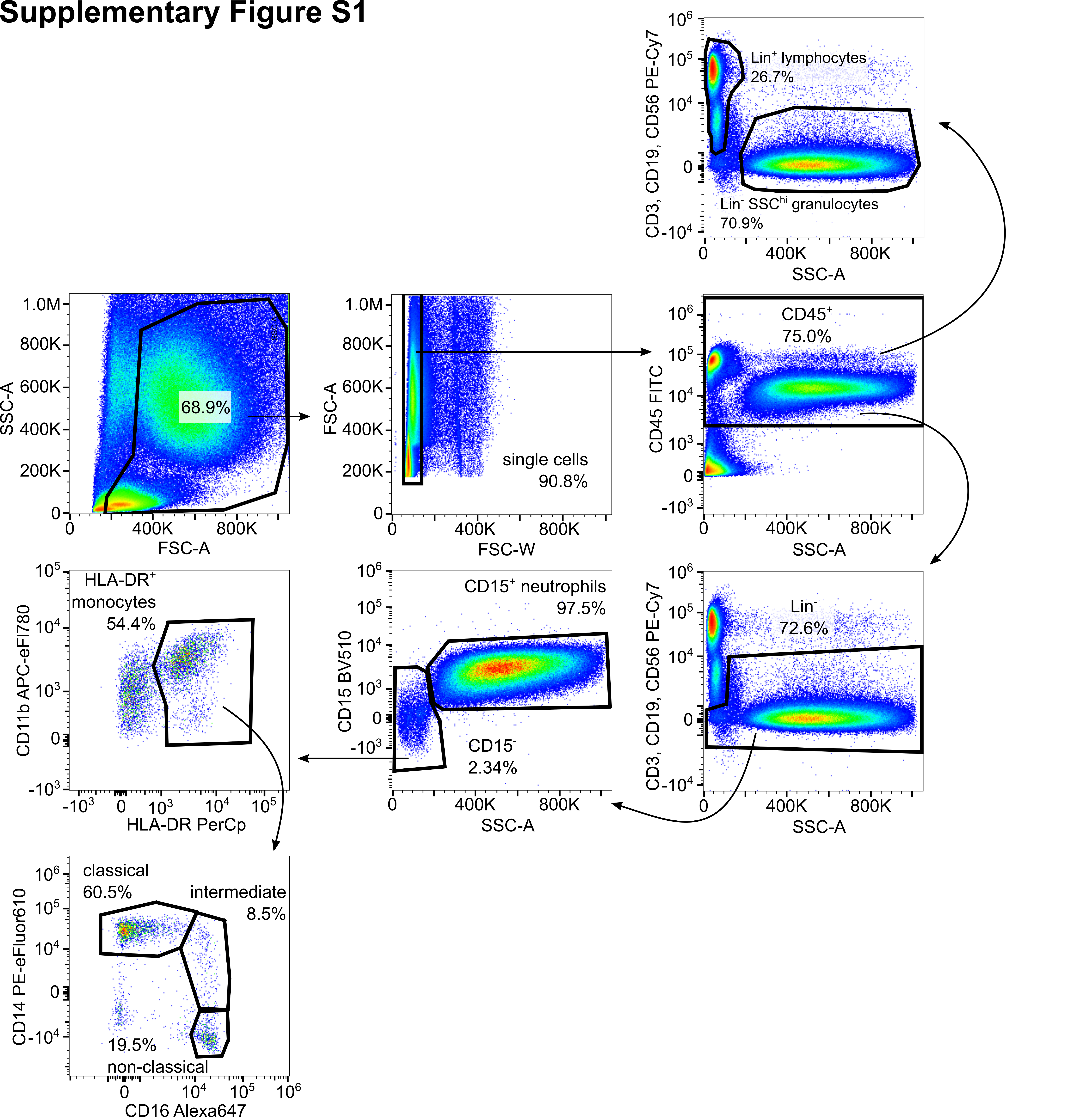

Supplement: Supplementary file 1 [file Image_1.TIFF]

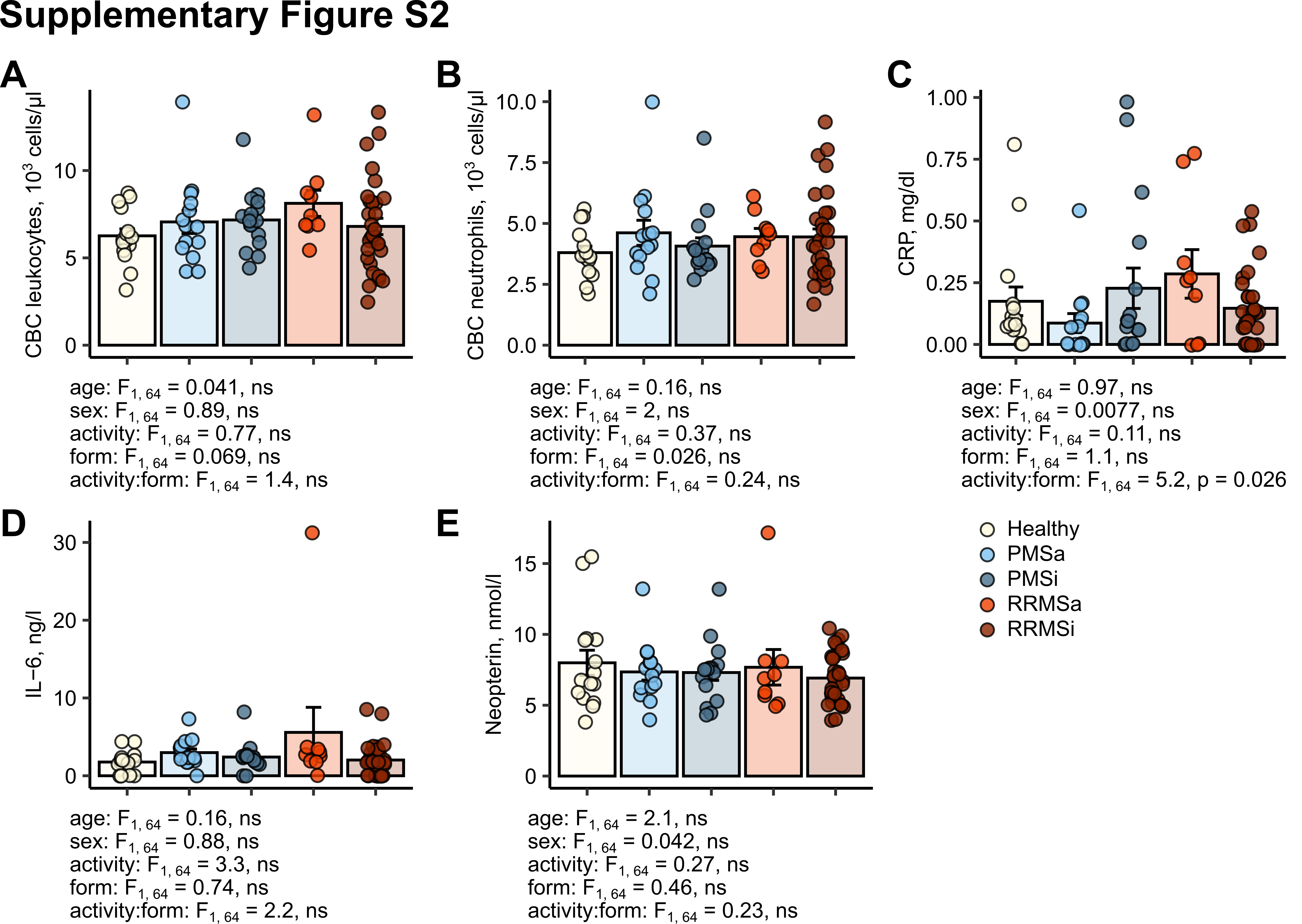

Supplement: Supplementary file 2 [file Image_2.TIFF]

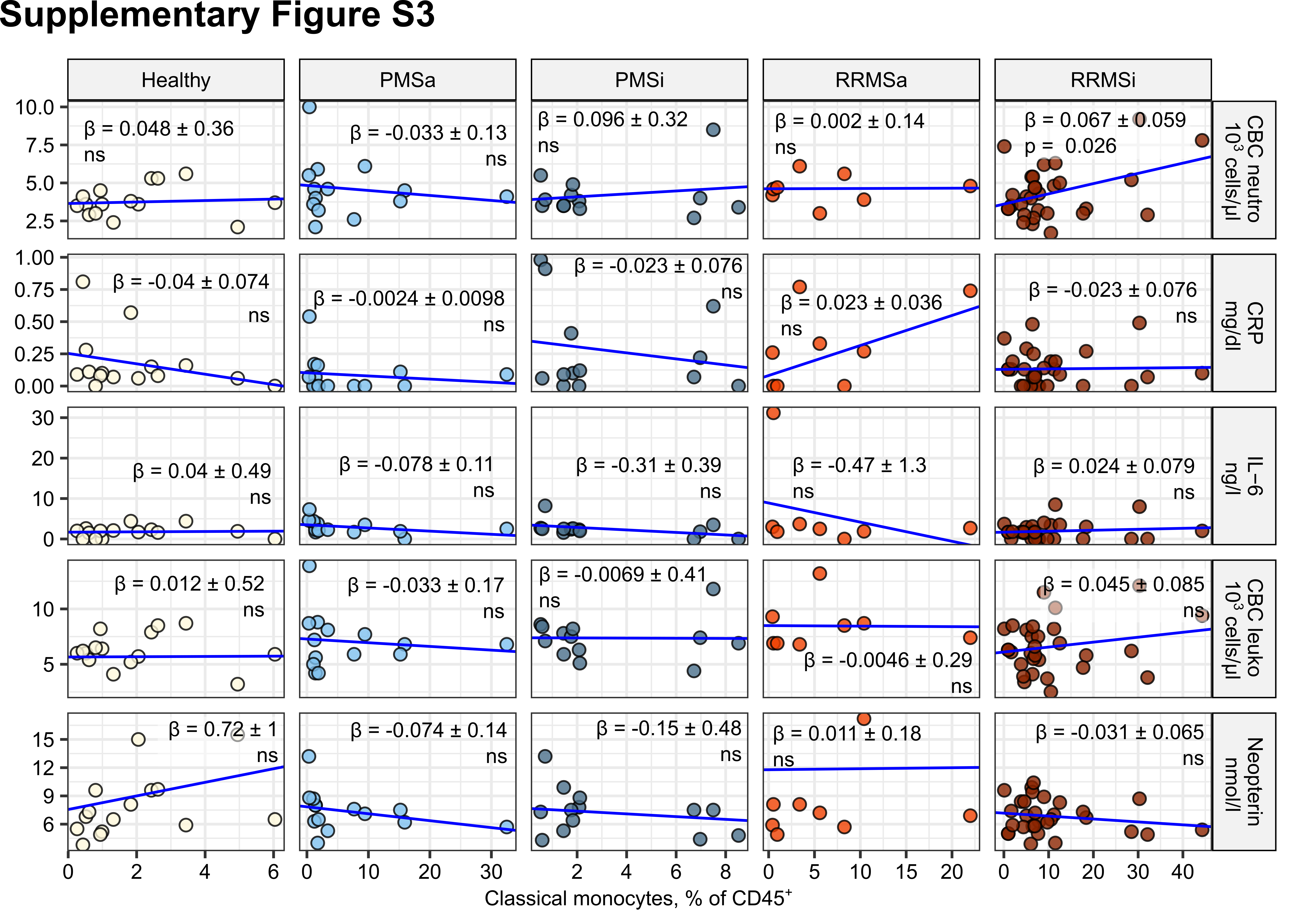

Supplement: Supplementary file 3 [file Image_3.TIFF]

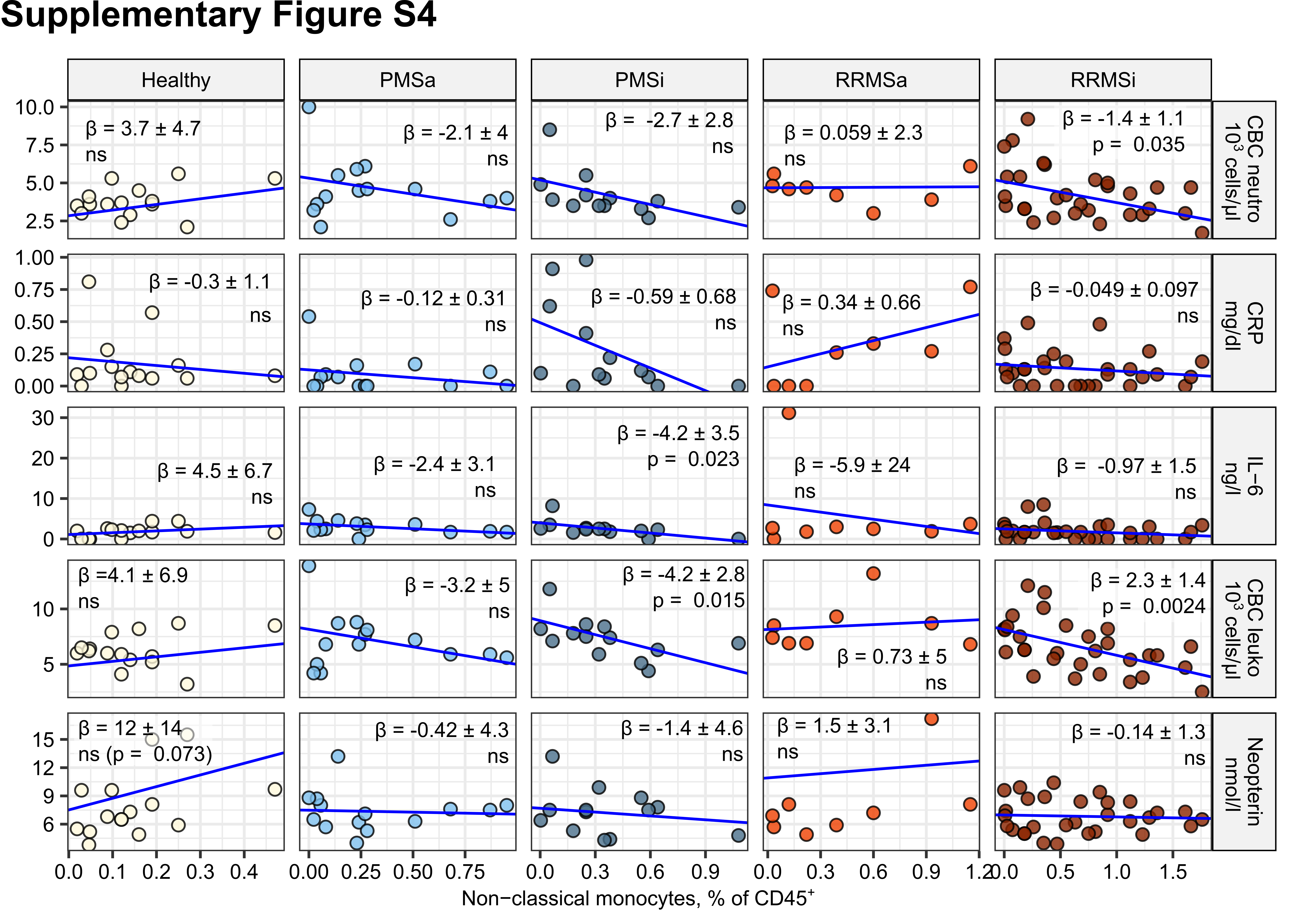

Supplement: Supplementary file 4 [file Image_4.TIFF]

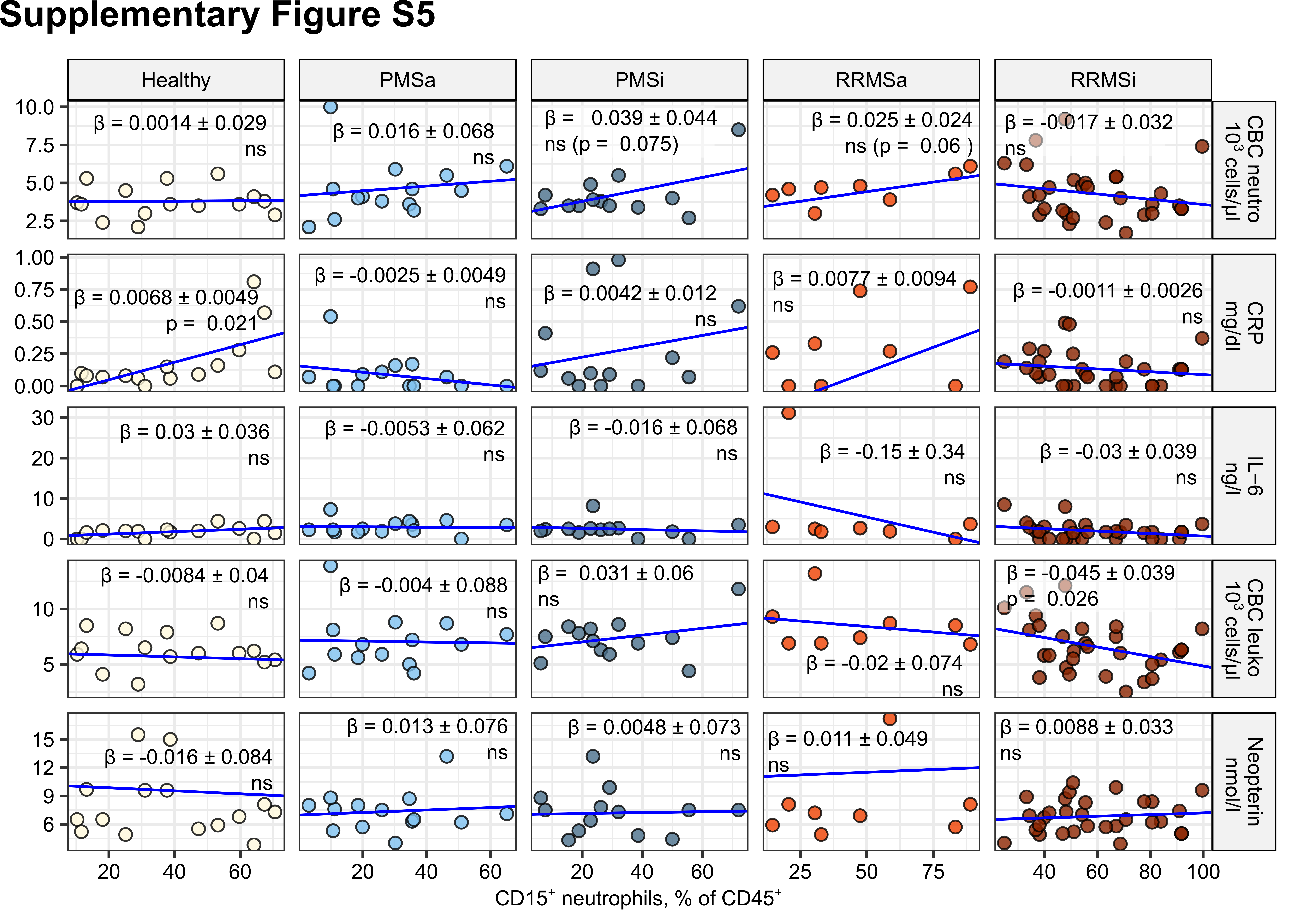

Supplement: Supplementary file 5 [file Image_5.TIFF]

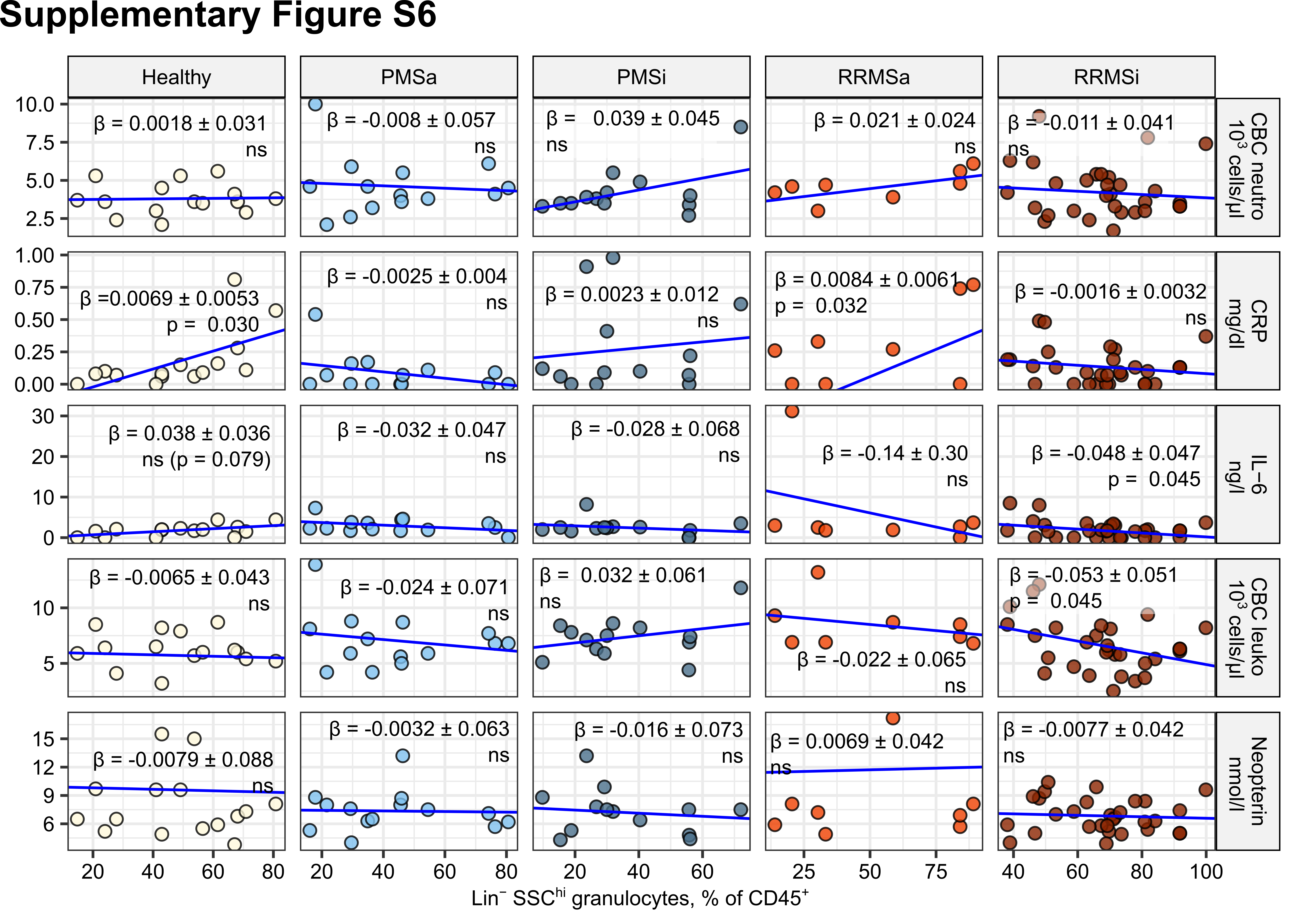

Supplement: Supplementary file 6 [file Image_6.TIFF]

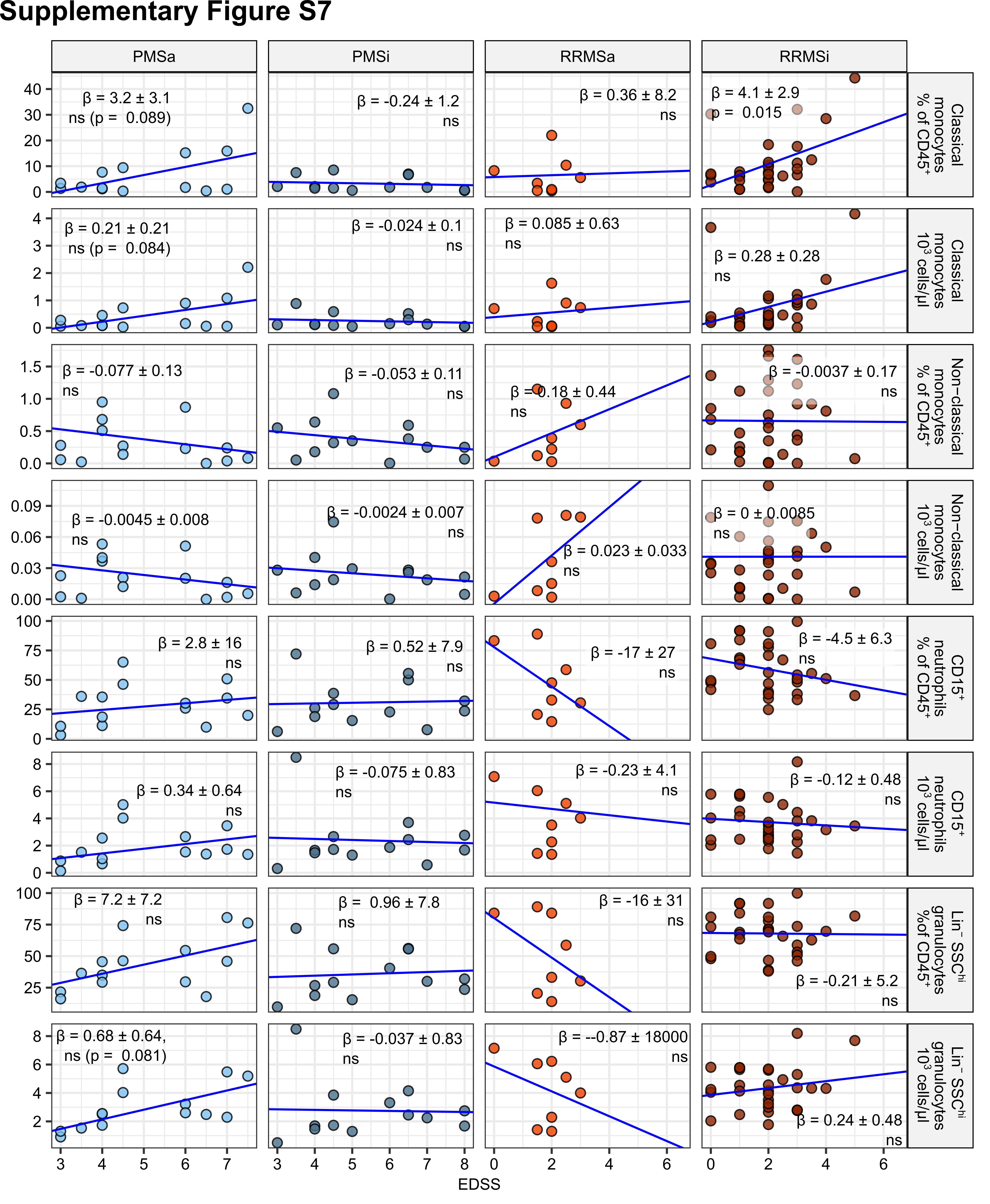

Supplement: Supplementary file 7 [file Image_7.TIFF]

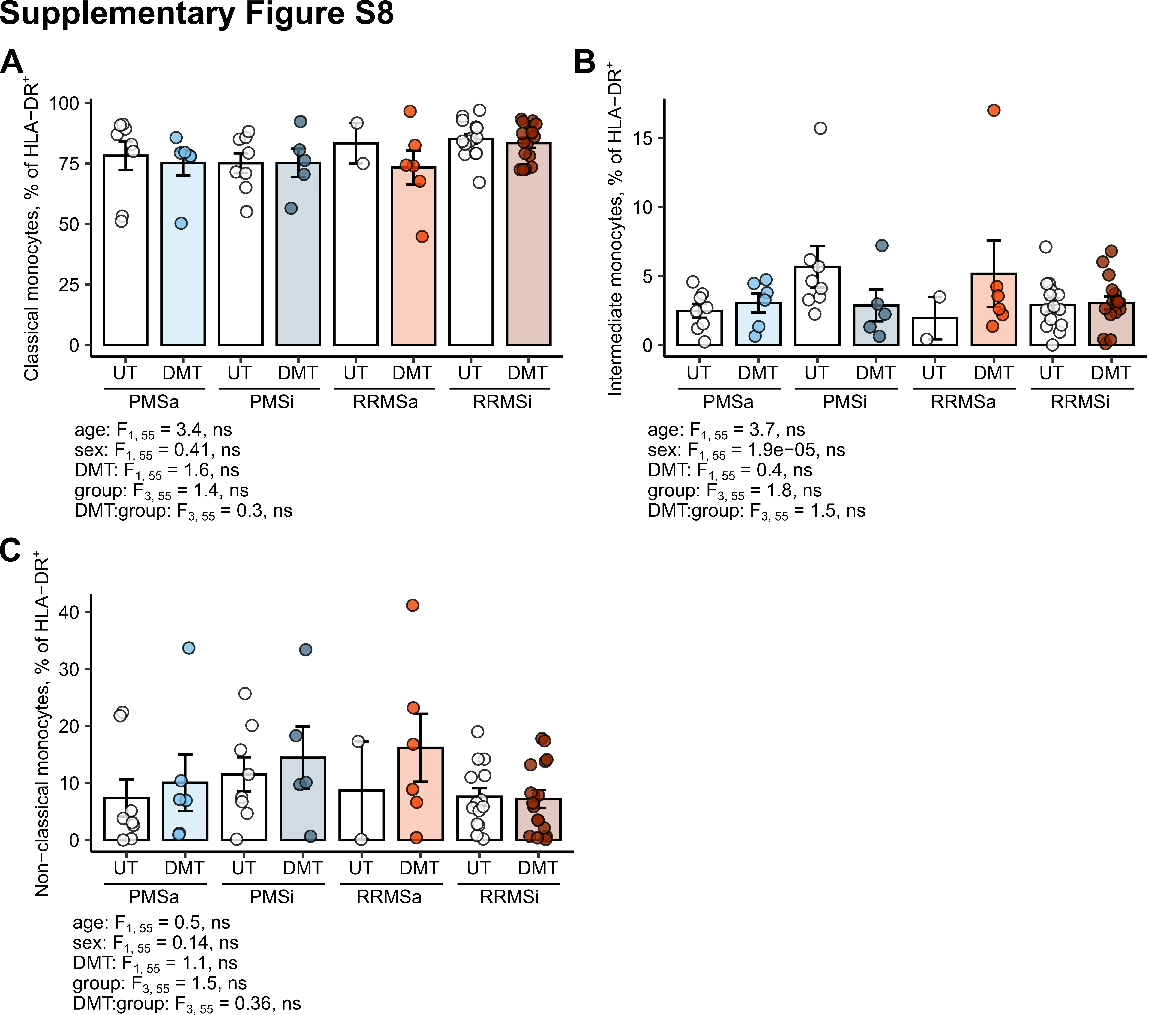

Supplement: Supplementary file 8 [file Image_8.TIFF]

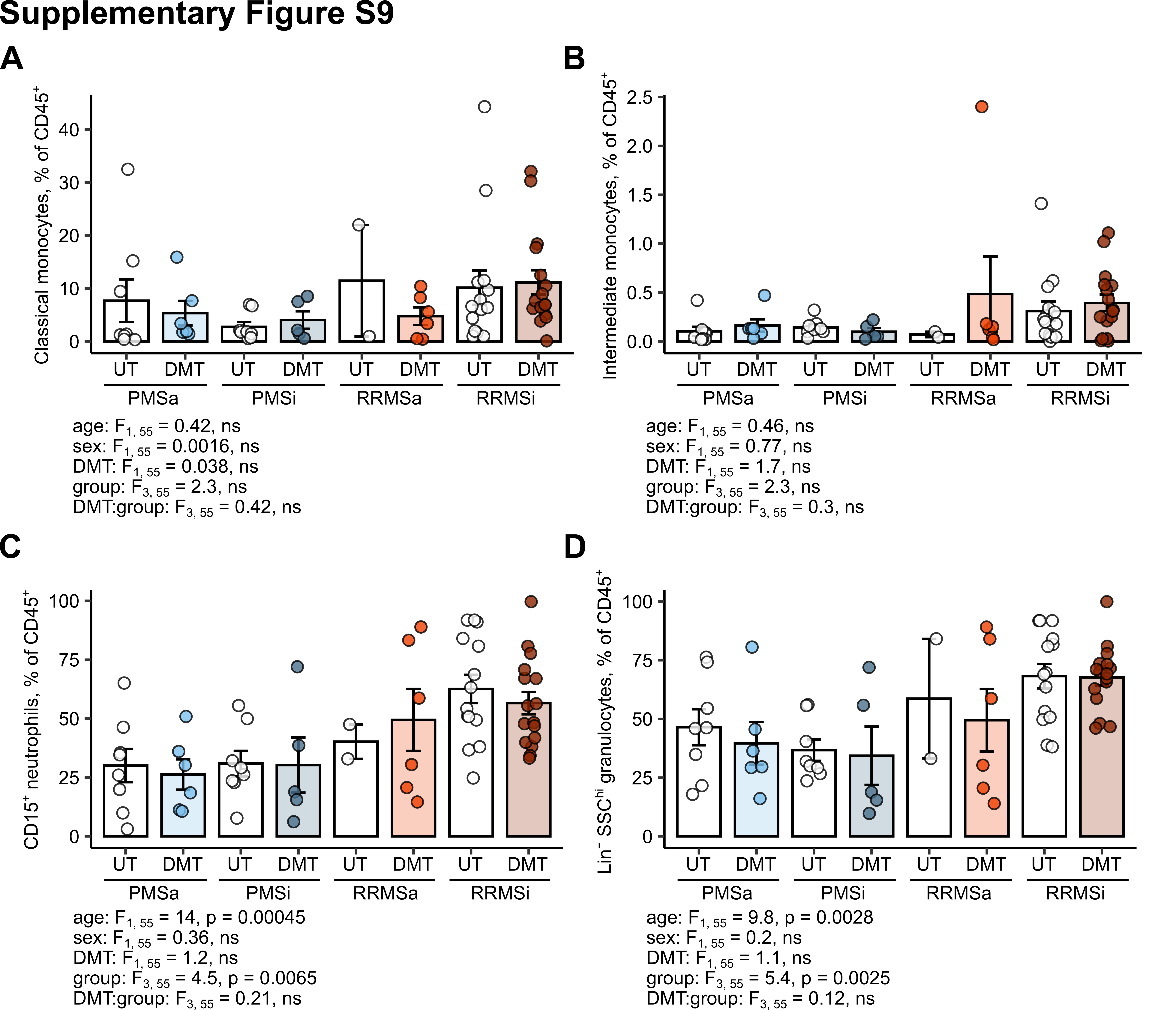

Supplement: Supplementary file 9 [file Image_9.TIFF]

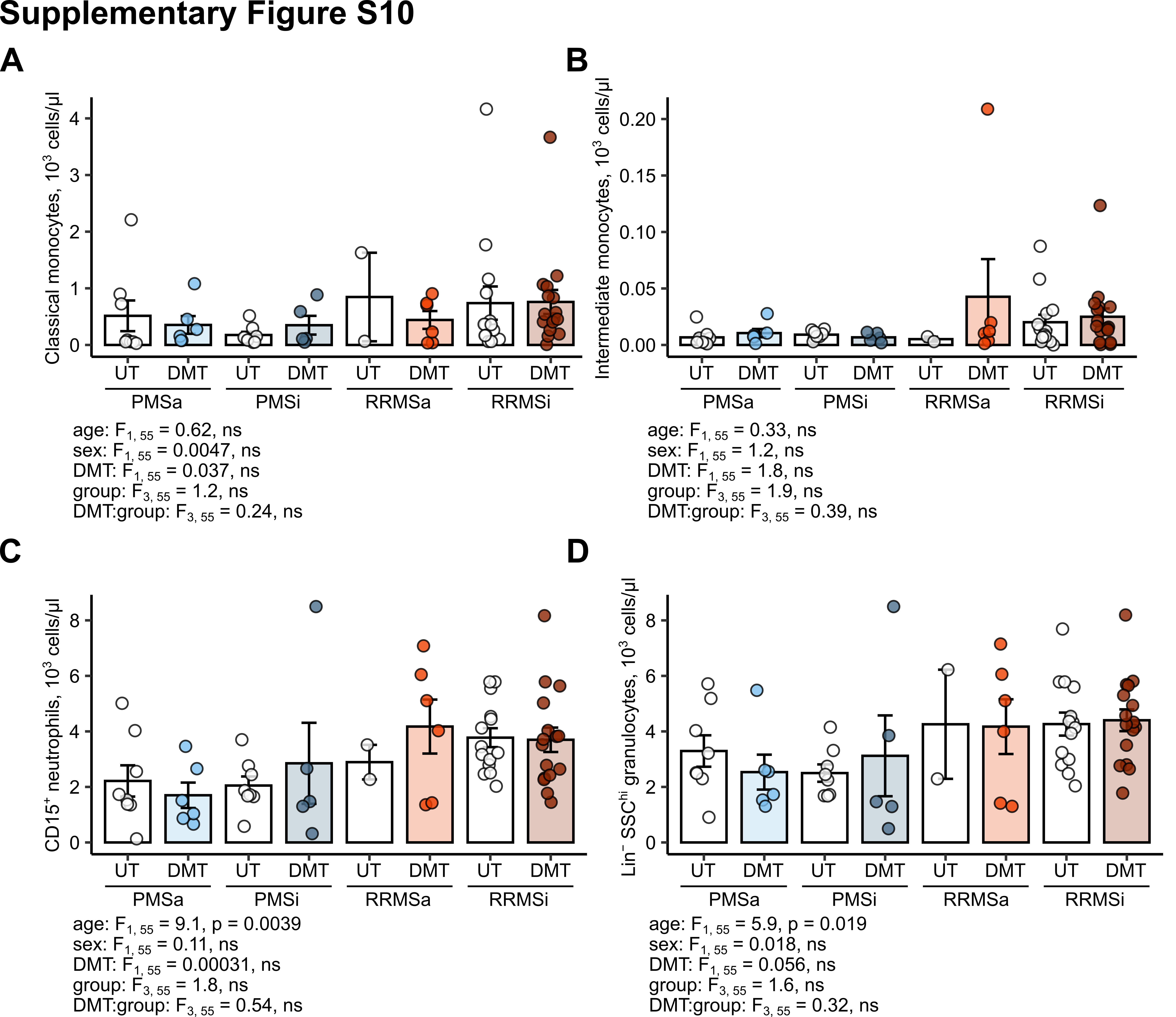

Supplement: Supplementary file 10 [file Image_10.TIFF]

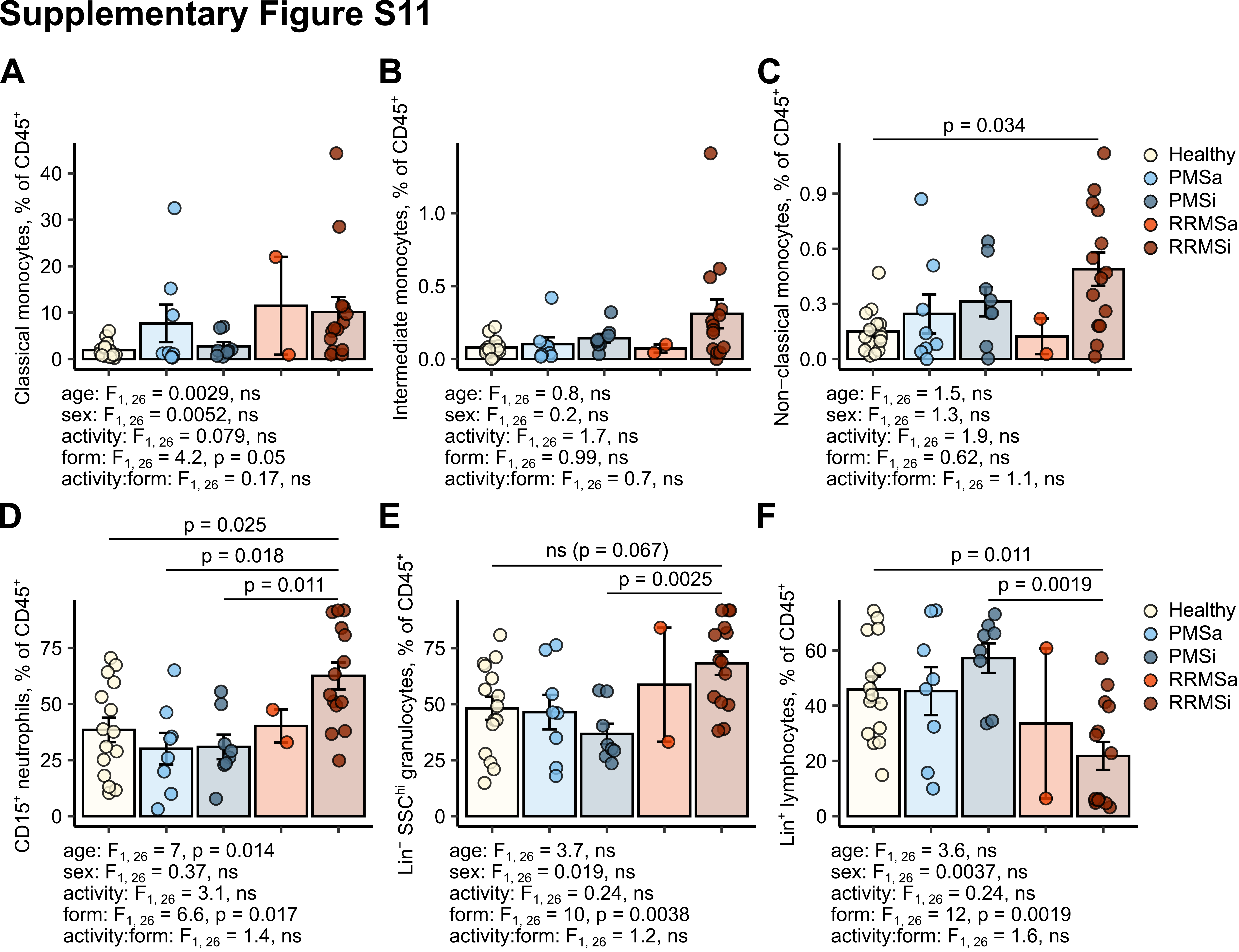

Supplement: Supplementary file 11 [file Image_11.TIFF]

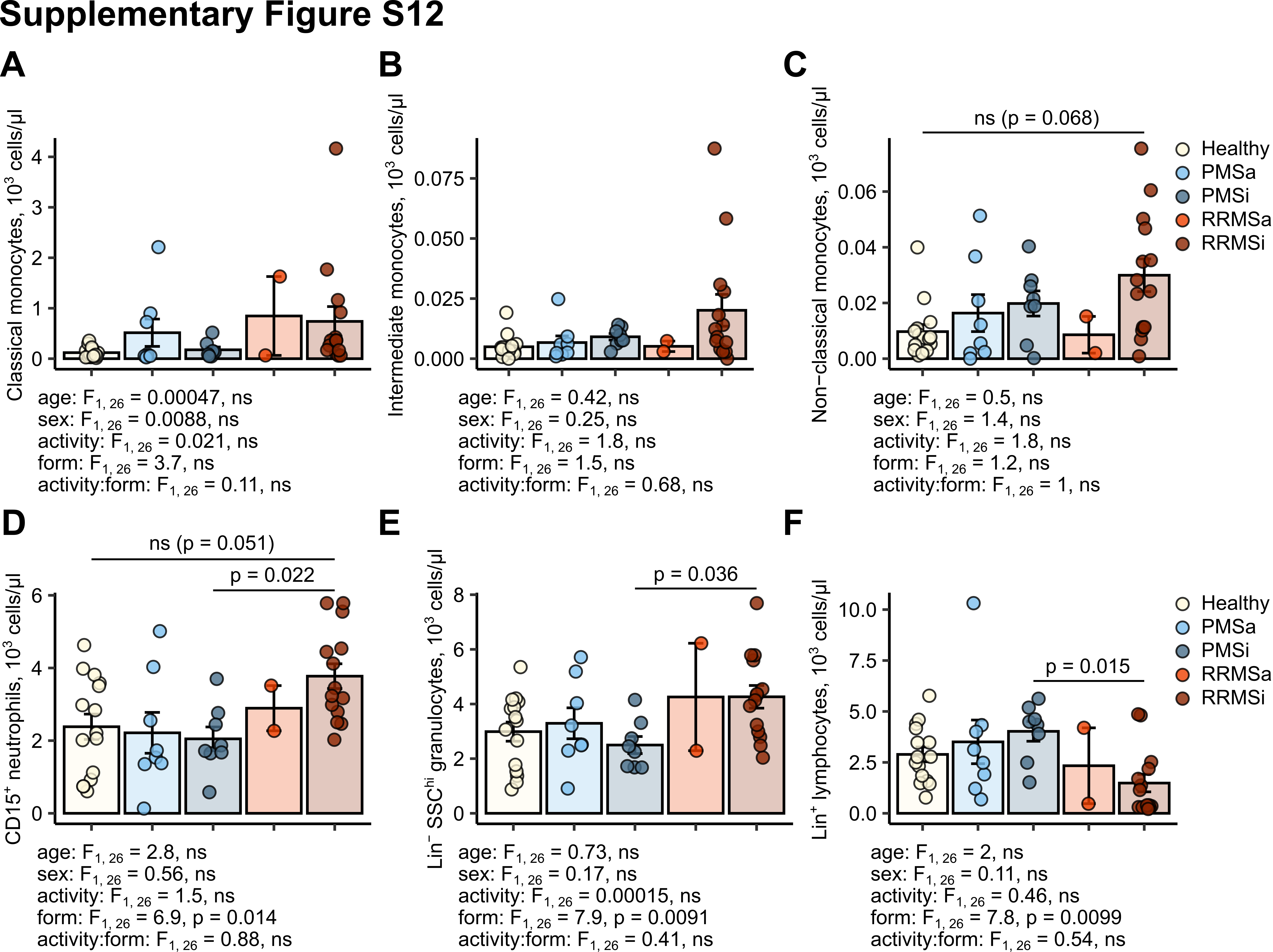

Supplement: Supplementary file 12 [file Image_12.TIFF]

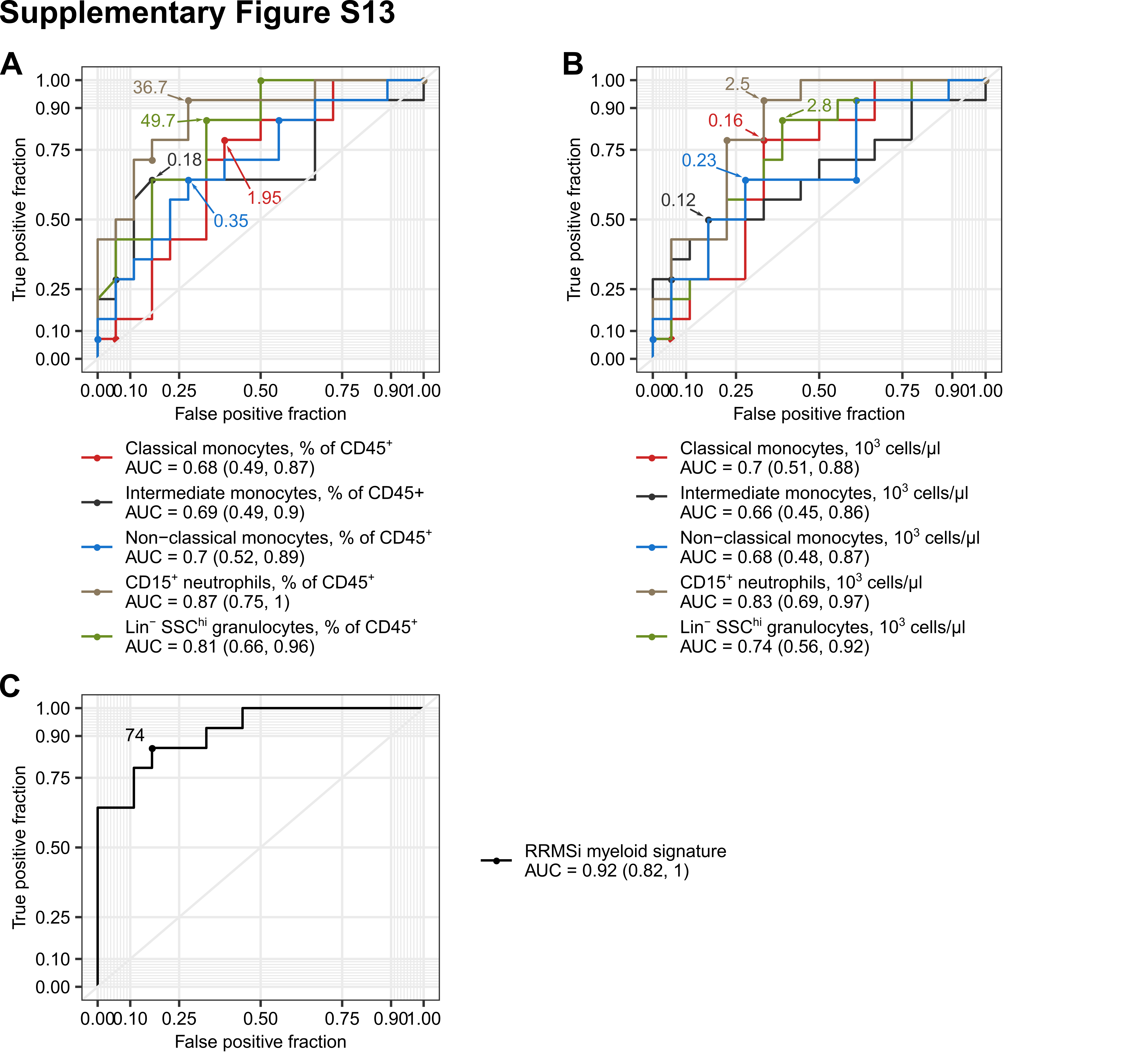

Supplement: Supplementary file 13 [file Image_13.TIFF]
