# Supplementary material for: Expansion of Neutrophils and Classical and Nonclassical Monocytes as a Hallmark in Relapsing-Remitting Multiple Sclerosis
Source: Front Immunol. 2020 Apr 29;11:594. doi: 10.3389/fimmu.2020.00594 (PMC7202453; doi:10.3389/fimmu.2020.00594)
Supplement: Supplementary file 14 [file Table_1.DOCX]

## Supplementary Table S1

Characteristics of study groups (Healthy – healthy control, PMSa – active Progressive MS, PMSi – inactive Progressive MS, RRMSa – active Relapsing-Remitting MS, RRMSi – inactive Relapsing-Remitting MS). ^1^ Expanded Disability Status Scale, ^2^ Relapse within the last 3 months before recruitment, ^3^ EDSS progression within the last 6 months before recruitment, ^4^ Disease-Modifying Therapy, ^5^ Chi^2^ test for all study groups, ^6^ one-way ANOVA for all study groups, ^7^ one-way ANOVA for MS groups, ^8^ Kruskal-Walis test for MS groups, ^9^ Chi^2^ test for MS groups.

|  | **Healthy** | **PMSa** | **PMSi** | **RRMSa** | **RRMSi** | **p** |
| --- | --- | --- | --- | --- | --- | --- |
| N, total | 15 | 14 | 16 | 9 | 31 | NA |
| N, with complete cytometry data | 15 | 14 | 13 | 8 | 30 | NA |
| Female  (number, percent) | 8 (53.3) | 10 (71.4) | 10 (62.5) | 7 (77.8) | 26 (83.9) | 0.92 ^5^ |
| Age, years  (mean, SD) | 32 (12) | 52 (7) | 53 (9.4) | 41 (9.1) | 39 (9) | < 0.0001 ^6^ |
| Disease duration, years  (mean, SD) | NA | 19.0 (8.7) | 22.8 (8.5) | 11.6 (5.8) | 12.6 (6.7) | 0.003 ^7^ |
| EDSS^1^  (median, range) | NA | 4.5 (3 – 7.5) | 4.5 (3 – 8.0) | 2.0 (0 – 6.5) | 2.0 (0 – 6.5) | < 0.0001 ^8^ |
| Recent relapse^2^  (number, percent) | NA | 3 (21.4) | 0 (0) | 9 (100) | 0 (0) | < 0.0001 ^9^ |
| EDSS progression^3^  (number, percent) | NA | 14 (100) | 0 (0) | 6 (66.7) | 0 (0) | < 0.0001 ^9^ |
| DMT received^4^  (number, percent): | NA | 6 (42.9) | 6 (37.5) | 6 (66.7) | 18 (58.1) | 0.81 ^9^ |
| Glatiramer acetate | NA | 0 (0) | 0 (0) | 1 (11.1) | 4 (12.9) | 0.33 ^9^ |
| Dimethyl fumarate | NA | 0 (0) | 0 (0) | 2 (22.2) | 5 (16.1) | 0.17 ^9^ |
| Fingolimod | NA | 0 (0) | 0 (0) | 1 (11.1) | 1 (3.23) | 0.4 ^9^ |
| Natalizumab | NA | 0 (0) | 6 (37.5) | 2 (22.2) | 8 (25.8) | 0.24 ^9^ |
| Rituximab | NA | 6 (42.9) | 0 (0) | 0 (0) | 0 (0) | 0.00022 ^9^ |
