# Supplementary material for: Expansion of Neutrophils and Classical and Nonclassical Monocytes as a Hallmark in Relapsing-Remitting Multiple Sclerosis
Source: Front Immunol. 2020 Apr 29;11:594. doi: 10.3389/fimmu.2020.00594 (PMC7202453; doi:10.3389/fimmu.2020.00594)
Supplement: Supplementary file 15 [file Table_2.DOCX]

## Supplementary Table S2

Results of ROC analysis for candidate RRMSi markers and Myeloid Signature in the entire MS participant collective (Figure 6) and DMT-untreated MS participant collective (Supplementary Figure S13). Optimal cutoff – optimal cutoff determined with Youden’s method, Se – sensitivity at cutoff, Sp – specificity at cutoff, AUC – area under the curve.

| **Study collective** | **Marker** | **Optimal cutoff** | **Se** | **Sp** | **AUC (95% CI)** |
| --- | --- | --- | --- | --- | --- |
| MS participants | Classical monocytes, % of CD45+ | 3.85 | 0.83 | 0.63 | 0.7 (0.57, 0.83) |
|  | Intermediate monocytes, % of CD45+ | 0.2 | 0.67 | 0.86 | 0.7 (0.55, 0.84) |
|  | Non-classical monocytes, % of CD45+ | 0.63 | 0.5 | 0.8 | 0.66 (0.52, 0.8) |
|  | CD15+ neutrophils, % of CD45+ | 36.7 | 0.9 | 0.69 | 0.82 (0.72, 0.92) |
|  | Lin- SSChi granulocytes, % of CD45+ | 46.1 | 0.93 | 0.63 | 0.8 (0.69, 0.91) |
|  | Classical monocytes, 103 cells/µl | 0.16 | 0.87 | 0.57 | 0.68 (0.55, 0.81) |
|  | Intermediate monocytes, 103 cells/µl | 0.01 | 0.63 | 0.83 | 0.67 (0.52, 0.81) |
|  | Non-Classical monocytes, 103 cells/µl | 0.02 | 0.7 | 0.6 | 0.62 (0.48, 0.76) |
|  | CD15+ neutrophils, 103 cells/µl | 1.77 | 0.97 | 0.51 | 0.74 (0.62, 0.86) |
|  | Lin- SSChi granulocytes, 103 cells/µl | 2.65 | 0.9 | 0.57 | 0.72 (0.6, 0.85) |
|  | Myeloid Signature | 57.9 | 0.87 | 0.74 | 0.85 (0.76, 0.94) |
| DMT-untreated MS participants | Classical monocytes, % of CD45+ | 1.95 | 0.79 | 0.61 | 0.68 (0.49, 0.87) |
|  | Intermediate monocytes, % of CD45+ | 0.18 | 0.64 | 0.83 | 0.69 (0.49, 0.9) |
|  | Non-classical monocytes, % of CD45+ | 0.35 | 0.64 | 0.72 | 0.7 (0.52, 0.89) |
|  | CD15+ neutrophils, % of CD45+ | 36.7 | 0.93 | 0.72 | 0.87 (0.75, 1) |
|  | Lin- SSChi granulocytes, % of CD45+ | 49.7 | 0.86 | 0.67 | 0.81 (0.66, 0.96) |
|  | Classical monocytes, 103 cells/µl | 0.17 | 0.79 | 0.67 | 0.7 (0.51, 0.88) |
|  | Intermediate monocytes, 103 cells/µl | 0.01 | 0.5 | 0.83 | 0.66 (0.45, 0.86) |
|  | Non-Classical monocytes, 103 cells/µl | 0.02 | 0.64 | 0.72 | 0.68 (0.48, 0.87) |
|  | CD15+ neutrophils, 103 cells/µl | 2.46 | 0.93 | 0.67 | 0.83 (0.69, 0.97) |
|  | Lin- SSChi granulocytes, 103 cells/µl | 2.79 | 0.86 | 0.61 | 0.74 (0.56, 0.92) |
|  | Myeloid Signature | 74 | 0.86 | 0.83 | 0.92 (0.82, 1) |
