# Supplementary material for: Expansion of Neutrophils and Classical and Nonclassical Monocytes as a Hallmark in Relapsing-Remitting Multiple Sclerosis
Source: Front Immunol. 2020 Apr 29;11:594. doi: 10.3389/fimmu.2020.00594 (PMC7202453; doi:10.3389/fimmu.2020.00594)
Supplement: Supplementary file 16 [file Table_3.DOCX]

## Supplementary Table S3

Results of Likelihood Ratio Tests for the logistic regression model used for development of the RRMSi myeloid signature in the entire MS participant collective (Figure 6C) and DMT-untreated MS participant collective (Supplementary Figure S13). Full model: model including percentage of classical monocytes, non-classical monocytes and CD15^+^ neutrophils within CD45^+^ cells. Df – degrees of freedom, Δ Deviance – difference in residual deviance between the full model and the model being tested, Δ Df – difference in degrees of freedom between the full model and the model being tested. Statistical significance (Δ Deviance ≠ 0) was assessed with Chi^2^ test.

| **Study collective** | **Model** | **Residual Df** | **Residual Deviance** | **ΔDf** | **ΔDeviance** | **P value** |
| --- | --- | --- | --- | --- | --- | --- |
| MS participants | Full: classical monocytes, non-classical monocytes, CD15+ neutrophils | 61 | 59 | NA | NA | NA |
|  | Null | 64 | 90 | -3 | -31 | < 0.0001 |
|  | Single term: classical monocytes | 63 | 84 | -2 | -25 | < 0.0001 |
|  | Single term: non-classical monocytes | 63 | 82 | -2 | -23 | < 0.0001 |
|  | Single term: CD15+ neutrophils | 63 | 69 | -2 | -11 | 0.0051 |
| DMT-untreated MS participants | Full: classical monocytes, non-classical monocytes, CD15+ neutrophils | 28 | 23 | NA | NA | NA |
|  | Null | 31 | 44 | -3 | -21 | 0.00011 |
|  | Single term: classical monocytes | 30 | 43 | -2 | -19 | < 0.0001 |
|  | Single term: non-classical monocytes | 30 | 39 | -2 | -16 | 0.00029 |
|  | Single term: CD15+ neutrophils | 30 | 28 | -2 | -5.3 | 0.072 |
