# Supplementary material for: Expansion of Neutrophils and Classical and Nonclassical Monocytes as a Hallmark in Relapsing-Remitting Multiple Sclerosis
Source: Front Immunol. 2020 Apr 29;11:594. doi: 10.3389/fimmu.2020.00594 (PMC7202453; doi:10.3389/fimmu.2020.00594)
Supplement: Supplementary file 18 [file Data_Sheet_1.PDF]

Supplementary Material

1 Supplementary Tables

1.1 Supplementary Table S1

Characteristics of study groups (Healthy – healthy control, PMSa – active Progressive MS, PMSi – inactive Progressive MS, RRMSa – active Relapsing-Remitting MS, RRMSi – inactive Relapsing-Remitting MS). [1] Expanded Disability Status Scale, [2] Relapse within the last 3 months before recruitment, [3] EDSS progression within the last 6 months before recruitment, [4] Disease-Modifying Therapy, [5] Chi^2 test for all study groups, [6] one-way ANOVA for all study groups, [7] one-way ANOVA for MS groups, [8] Kruskal-Walis test for MS groups, [9] Chi^2 test for MS groups.

1.2 Supplementary Table S2

Results of ROC analysis for candidate RRMSi markers and Myeloid Signature in the entire MS participant collective (Figure 6) and DMT-untreated MS participant collective (Supplementary Figure S13). Optimal cutoff – optimal cutoff determined with Youden's method, Se – sensitivity at cutoff, Sp – specificity at cutoff, AUC – area under the curve.

1.3 Supplementary Table S3

Results of Likelihood Ratio Tests for the logistic regression model used for development of the RRMSi myeloid signature in the entire MS participant collective (Figure 6C) and DMT-untreated MS participant collective (Supplementary Figure S13). Full model: model including percentage of classical monocytes, non-classical monocytes and $CD15^+$ neutrophils within $CD45^+$ cells. Df – degrees of freedom, Δ Deviance – difference in residual deviance between the full model and the model being tested, Δ Df – difference in degrees of freedom between the full model and the model being tested. Statistical significance (Δ Deviance ≠ 0) was assessed with Chi^2 test.

1.4 Supplementary Table S4

Data analyzed in the current study.

2 Supplementary Figures

Supplementary Figure S1. Flow cytometry data analysis scheme for immunophenotyping of blood leukocytes. Lin^+ lymphocytes are defined as $CD45^+$ $CD3^+$ or $CD19^+$ or $CD56^+$ cells, Lin^- SSC^{hi} granulocytes as $CD45^+$ $CD3^-$ $CD19^-$ $CD56^-$ SSC^{hi} cells. $CD15^+$ neutrophils are defined as $CD45^+$ $CD3^-$ $CD19^-$ $CD56^-$ $CD15^+$ SSC^{hi} cells, classical monocytes as $CD45^+$ $CD3^-$ $CD19^-$ $CD56^-$ $CD15^-$ HLA-DR^+ $CD14^{++}$ $CD16^-$, intermediate monocytes as $CD45^+$ $CD3^-$ $CD19^-$ $CD56^-$ $CD15^-$ HLA-DR^+ $CD14^{++}$ $CD16^+$, non-classical monocytes as $CD45^+$ $CD3^-$ $CD19^-$ $CD56^-$ $CD15^-$ HLA-DR^+ $CD14^+$ $CD16^+$ cells.

Supplementary Figure S2. Readouts of systemic inflammation in healthy controls and MS course types. Blood leukocyte and neutrophil count determined by standard complete blood count (CBC) measurement, serum concentrations of C-reactive protein (CRP), interleukin 6 (IL-6) and neopterin in healthy controls (n = 15) and MS patients stratified by disease course type (PMSa – PMS active: n = 14, PMSi – PMS inactive: n = 16, RRMSa – RRMS active: n = 9 and RRMSi – RRMS inactive: n = 31). Each point denotes a single observation, bars depict group-wise means, error bars represent SEM. Statistical significance was determined by one (healthy/MS disease status) and two-way (disease progression form, activity and form: activity interaction, MS collective) ANCOVA with age and sex as confounders. Results of the two-way ANCOVA are presented under the plots. Post-hoc testing was performed with Benjamini-Hochberg-corrected two-tailed T tests. Significant results of post-hoc test are presented within the plots. (**A**) Total blood leukocytes. ANCOVA for the disease status: $F_{1, 81} = 1.6$, ns, age: $F_{1, 81} = 0.0049$, ns, sex: $F_{1, 81} = 0.67$, ns. (**B**) Total blood neutrophils. ANCOVA for the disease status: $F_{1, 81} = 1.7$, ns, age: $F_{1, 81} = 0.12$, ns, sex: $F_{1, 81} = 2.1$, ns. (**C**) Serum CRP. ANCOVA for the disease status: $F_{1, 81} = 0.065$, ns, age: $F_{1, 81} = 0.19$, ns, sex: $F_{1, 81} = 0.0056$, ns. (**D**) Serum IL-6. ANCOVA for the disease status: $F_{1, 81} = 0.19$, ns, age: $F_{1, 81} = 0.7$, ns, sex: $F_{1, 81} = 0.98$, ns. (**E**) Serum neopterin. ANCOVA for the disease status: $F_{1, 81} = 3.7$, ns, age: $F_{1, 81} = 3.6$, ns, sex: $F_{1, 81} = 1.5$, ns.

Supplementary Figure S3. Correlations of classical monocyte levels with readouts of systemic inflammation. Levels of classical monocytes expressed as percent of $CD45^+$ blood cells were correlated with neutrophil and leukocytes counts determined by standard complete blood count (CBC) measurement, serum concentrations of C-reactive protein (CRP), interleukin 6 (IL-6) and neopterin in each study group (healthy controls: n = 15, PMSa – PMS active: n = 14, PMSi – PMS inactive: n = 13, RRMSa – RRMS active: n = 8 and RRMSi – RRMS inactive: n = 30). Correlations were performed with mixed-effect linear models with age and sex adjustment. Each point denotes single observation, blue lines depict fitted regression trends. Estimates for regression trend slope (β) with 95% CI and p values ($\beta \neq 0$, two-tailed T test) are shown.

Supplementary Figure S4. Correlations of non-classical monocyte levels with readouts of systemic inflammation. Levels of non-classical monocytes expressed as percent of $CD45^+$ blood cells were correlated with neutrophil and leukocytes counts determined by standard complete blood count (CBC) measurement, serum concentrations of C-reactive protein (CRP), interleukin 6 (IL-6) and neopterin in each study group (healthy controls: n = 15, PMSa – PMS active: n = 14, PMSi – PMS inactive: n = 13, RRMSa – RRMS active: n = 8 and RRMSi – RRMS inactive: n = 30). Correlations were performed with mixed-effect linear models with age and sex adjustment. Each point denotes single observation, blue lines depict fitted regression trends. Estimates for regression trend slope (β) with 95% CI and p values ($\beta \neq 0$, two-tailed T test) are shown.

Supplementary Figure S5. Correlations of $CD15^+$ neutrophil levels with readouts of systemic inflammation. Levels of $CD15^+$ neutrophils expressed as percent of $CD45^+$ blood cells were correlated with neutrophil and leukocytes counts determined by standard complete blood count (CBC) measurement, serum concentrations of C-reactive protein (CRP), interleukin 6 (IL-6) and neopterin in each study group (healthy controls: n = 15, PMSa – PMS active: n = 14, PMSi – PMS inactive: n = 13, RRMSa – RRMS active: n = 8 and RRMSi – RRMS inactive: n = 30). Correlations were performed with mixed-effect linear models with age and sex adjustment. Each point denotes single observation, blue lines depict fitted regression trends. Estimates for regression trend slope (β) with 95% CI and p values ($\beta \neq 0$, two-tailed T test) are shown.

Supplementary Figure S6. Correlations of Lin^- SSC^{hi} granulocyte levels with readouts of systemic inflammation. Levels of Lin^- SSC^{hi} grynulocytes expressed as percent of $CD45^+$ blood cells were correlated with neutrophil and leukocytes counts determined by standard complete blood count (CBC) measurement, serum concentrations of C-reactive protein (CRP), interleukin 6 (IL-6) and neopterin in each study group (healthy controls: n = 15, PMSa – PMS active: n = 14, PMSi – PMS inactive: n = 13, RRMSa – RRMS active: n = 8 and RRMSi – RRMS inactive: n = 30). Correlations were performed with mixed-effect linear models with age and sex adjustment. Each point denotes single observation, blue lines depict fitted regression trends. Estimates for regression trend slope (β) with 95% CI and p values ($\beta \neq 0$, two-tailed T test) are shown.

Supplementary Figure S7. Correlations of myeloid cell levels with EDSS. Percentages and absolute counts of $CD15^+$ neutrophils, classical and non-classical monocytes and Lin^- SSC^{hi} granulocytes were correlated with expanded disability status scale (EDSS) in each MS study group (PMSa – PMS active: n = 14, PMSi – PMS inactive: n = 13, RRMSa – RRMS active: n = 8 and RRMSi – RRMS inactive: n = 30). Correlations were performed with mixed-effect linear models with age and sex adjustment. Each point denotes single observation, blue lines depict fitted regression trends. Estimates for regression trend slope (β) with 95% CI and p values ($\beta \neq 0$, two-tailed T test) are shown.

Supplementary Figure S8. No effect of disease-modifying therapy on monocyte subpopulation distribution pattern. Classical, intermediate and non-classical monocytes were identified as presented in Supplementary Figure S1 in blood samples of MS patients, for whom data on disease-modifying therapy (untreated: UT, disease-modifying therapy: DMT) were available (UT PMSa – PMS active: n = 8, DMT PMSa – PMS active: n = 6, UT PMSi – PMS inactive: n = 8, DMT PMSi – PMS inactive: n = 5, UT RRMSa – RRMS active: n = 5, DMT RRMSa – RRMS active: n = 6, UT RRMSi - RRMS inactive: n = 14, DMT RRMSi – RRMS inactive: n = 16). Levels of the studied populations are expressed as percentages of $HLA\text{-}DR^+$ pan-monocytes. Each point denotes a single observation, bars depict group-wise means, error bars represent SEM. Statistical significance was calculated with two-way ANCOVA (terms: study group, DMT and group: DMT interaction) with age and sex as confounders. Results of the two-way ANCOVA are presented under the plots. Post-hoc testing was performed with Benjamini-Hochberg-corrected two-tailed T tests. Significant results of post-hoc test are presented within the plots. (**A**) Classical monocytes, (**B**) intermediate monocytes, (**C**) non-classical monocytes.

Supplementary Figure S9. No effect of disease-modifying therapy on relative abundance of classical and intermediate monocytes, CD15$^+$ neutrophils and Lin$^-$ SSC^{hi} granulocytes. Classical and intermediate monocytes, CD15$^+$ neutrophils and Lin$^-$ SSC^{hi} granulocytes were identified in blood samples of MS patients, for whom data on disease-modifying therapy (untreated: UT, disease-modifying therapy: DMT) were available (UT PMSa – PMS active: n = 8, DMT PMSa – PMS active: n = 6, UT PMSi – PMS inactive: n = 8, DMT PMSi – PMS inactive: n = 5, UT RRMSa – RRMS active: n = 5, DMT RRMSa – RRMS active: n = 6, UT RRMSi – RRMS inactive: n = 14, DMT RRMSi – RRMS inactive: n = 16) as presented in Supplementary Figure S1. Levels of the studied populations are expressed as percentage of CD45$^+$ blood leukocytes. Each point denotes a single observation, bars depict group-wise means, error bars represent SEM. Statistical significance was calculated with two-way ANCOVA (terms: study group, DMT and group: DMT interaction) with age and sex as confounders. Results of the two-way ANCOVA are presented under the plots. Post-hoc testing was performed with Benjamini-Hochberg-corrected two-tailed T tests. Significant results of post-hoc test are presented within the plots. (**A**) Classical monocytes, (**B**) intermediate monocytes, (**C**) CD15$^+$ neutrophils, (**D**) Lin$^-$ SSC^{hi} granulocytes.

Supplementary Figure S10. No effect of disease-modifying therapy on counts of classical and intermediate monocytes, CD15$^+$ neutrophils and Lin$^-$ SSC^{hi} granulocytes. Classical and intermediate monocytes, CD15$^+$ neutrophils and Lin$^-$ SSC^{hi} granulocytes were identified in blood samples of MS patients, for whom data on disease-modifying therapy (untreated: UT, disease-modifying therapy: DMT) were available (UT PMSa – PMS active: n = 8, DMT PMSa – PMS active: n = 6, UT PMSi – PMS inactive: n = 8, DMT PMSi – PMS inactive: n = 5, UT RRMSa – RRMS active: n = 5, DMT RRMSa – RRMS active: n = 6, UT RRMSi – RRMS inactive: n = 14, DMT RRMSi – RRMS inactive: n = 16) as presented in Supplementary Figure S1. Levels of the studied populations are expressed as cell count per µl whole blood. Each point denotes a single observation, bars depict group-wise means, error bars represent SEM. Statistical significance was calculated with two-way ANCOVA (terms: study group, DMT and group: DMT interaction) with age and sex as confounders. Results of the two-way ANCOVA are presented under the plots. Post-hoc testing was performed with Benjamini-Hochberg-corrected two-tailed T tests. Significant results of post-hoc test are presented within the plots. (**A**) Classical monocytes, (**B**) intermediate monocytes, (**C**) CD15$^+$ neutrophils, (**D**) Lin$^-$ SSC^{hi} granulocytes.

Supplementary Figure S11. Changes in percentages of myeloid cell populations in DMT-untreated inactive relapsing-remitting MS individuals. Lin$^+$ lymphocytes, Lin$^-$ SSC^{hi} granulocytes, CD15$^+$ neutrophils and monocyte subpopulations were identified in whole-blood samples from healthy controls (n = 15) and MS patients stratified by disease course type (PMSa – PMS active: n = 8, PMSi – PMS inactive: n = 8, RRMSa – RRMS active: n = 2 and RRMSi – RRMS inactive: n = 14) as presented in Supplementary Figure S1. Levels of the studied populations are expressed as percentages of CD45$^+$ blood leukocytes. Each point denotes a single observation, bars depict group-wise means, error bars represent SEM. Statistical significance was determined by one (healthy/MS disease status) and two-way (disease progression form, activity and form: activity interaction, MS collective) ANCOVA with age and sex as confounders. Results of the two-way ANCOVA are presented under the plots. Post-hoc testing was performed with Benjamini-Hochberg-corrected two-tailed T tests. Significant results of post-hoc test are presented within the plots. (**A**) Classical monocytes. ANCOVA for the disease status: $F_{1, 43}$ = 3, ns, age: $F_{1, 43}$ = 1.4, ns, sex: $F_{1, 43}$ = 0.034, ns. (**B**) Intermediate monocytes. ANCOVA for the

disease status: $F_{1, 43}$ = 3.1, ns, age: $F_{1, 43}$ = 0.11, ns, sex: $F_{1, 43}$ = 0.63, ns. (**C**) Non-classical monocytes. ANCOVA for the disease status: $F_{1, 43}$ = 8.3, p = 0.062, age: $F_{1, 43}$ = 0.32, ns, sex: $F_{1, 43}$ = 0.45, ns. (**D**) CD15$^+$ neutrophils. ANCOVA for the disease status: $F_{1, 43}$ = 2.7, ns, age: $F_{1, 43}$ = 1.1, ns, sex: $F_{1, 43}$ = 2.9, ns. (**E**) Lin$^-$ SSC^{hi} granulocytes. ANCOVA for the disease status: $F_{1, 43}$ = 1.7, ns, age: $F_{1, 43}$ = 0.26, ns, $F_{1, 43}$ = 1, ns. (**F**) Lin$^+$ lymphocytes. ANCOVA for the disease status: $F_{1, 43}$ = 2.4, ns, age: $F_{1, 43}$ = 0.11, ns, sex: $F_{1, 43}$ = 0.76, ns.

Supplementary Figure S12. Changes in counts of myeloid cell populations in DMT-untreated inactive relapsing-remitting MS individuals. Lin$^+$ lymphocytes, Lin$^-$ SSC^{hi} granulocytes, CD15$^+$ neutrophils and monocyte subpopulations were identified in whole-blood samples from healthy controls (n = 15) and MS patients stratified by disease course type (PMSa – PMS active: n = 8, PMSi – PMS inactive: n = 8, RRMSa – RRMS active: n = 2 and RRMSi – RRMS inactive: n = 14) as presented in Supplementary Figure S1. Levels of the studied populations are expressed as count per µl whole blood. Each point denotes a single observation, bars depict group-wise means, error bars represent SEM. Statistical significance was determined by one (healthy/MS disease status) and two-way (disease progression form, activity and form: activity interaction, MS collective) ANCOVA with age and sex as confounders. Results of the two-way ANCOVA are presented under the plots. Post-hoc testing was performed with Benjamini-Hochberg-corrected two-tailed T tests. Significant results of post-hoc test are presented within the plots. (**A**) Classical monocytes. ANCOVA for the disease status: $F_{1, 43}$ = 2.4, ns, age: $F_{1, 43}$ = 0.95, ns, sex: $F_{1, 43}$ = 0.11, ns. (**B**) Intermediate monocytes. ANCOVA for the disease status: $F_{1, 43}$ = 3, ns, age: $F_{1, 43}$ = 0.11, ns, sex: $F_{1, 43}$ = 0.71, ns. (**C**) Non-classical monocytes. ANCOVA for the disease status: $F_{1, 43}$ = 7.6, p = 0.0085, age: $F_{1, 43}$ = 0.38, ns, sex: $F_{1, 43}$ = 0.78, ns. (**D**) CD15$^+$ neutrophils. ANCOVA for the disease status: $F_{1, 43}$ = 3, ns, age: $F_{1, 43}$ = 0.58, ns, sex: $F_{1, 43}$ = 3.1, ns. (**E**) Lin$^-$ SSC^{hi} granulocytes. ANCOVA for the disease status: $F_{1, 43}$ = 2.3, ns, age: $F_{1, 43}$ = 0.058, ns, sex: $F_{1, 43}$ = 1.1, ns. (**F**) Lin$^+$ lymphocytes. ANCOVA for the disease status: $F_{1, 43}$ = 0.18, ns, age: $F_{1, 43}$ = 0.024, ns, sex: $F_{1, 43}$ = 0.28, ns.

Supplementary Figure S13. Levels of neutrophils, classical and non-classical monocytes as RRMSi markers in DMT-untreated MS patients. Lin$^+$ lymphocytes, Lin$^-$ SSC^{hi} granulocytes, CD15$^+$ neutrophils and monocyte subpopulations were identified and quantified in DMT-untreated MS study participants stratified by disease course type (PMSa – PMS active: n = 8, PMSi – PMS inactive: n = 8, RRMSa – RRMS active: n = 2 and RRMSi – RRMS inactive: n = 14) as presented in Supplementary Figure S1. Myeloid signature was calculated as an optimally weighted sum of percentages of neutrophils, classical and non-classical monocytes within CD45$^+$ pan leukocytes. Receiver-Operator-Curves (ROC) display sensitivity and specificity of each parameter as a marker to differentiate between RRMSi and other MS disease courses. In the plots, optimal parameter cutoffs are displayed. In the legends, values of areas under the curve (AUC) for each ROC are presented with the 2.5% and 97.5% CI in parentheses. (**A**) Levels of myeloid cell populations and Lin$^+$ lymphocytes expressed as percentage of CD45$^+$ cells. (**B**) Levels of myeloid cell populations and Lin$^+$ lymphocytes expressed as count per µl whole blood. (**C**) Myeloid signature.
